# Structural basis for T-cell intracellular antigen-1 amyloid fibril formation revealed by cryo-electron microscopy

**DOI:** 10.1093/pnasnexus/pgaf388

**Published:** 2025-12-11

**Authors:** Daigo Inaoka, Tomoko Miyata, Fumiaki Makino, Yasuko Ohtani, Miu Ekari, Ryoga Kobayashi, Kayo Imamura, Emi Sakamoto, Takashi S Kodama, Norio Yoshida, Takayuki Kato, Keiichi Namba, Hidehito Tochio, Naotaka Sekiyama

**Affiliations:** Department of Biophysics, Graduate School of Science, Kyoto University, Kitashirakawa Oiwake-cho, Sakyo-ku, Kyoto, Kyoto 606-8502, Japan; Graduate School of Frontier Biosciences, The University of Osaka, 1-3 Yamadaoka, Suita, Osaka 565-0871, Japan; JEOL YOKOGUSHI Research Alliance Laboratories, The University of Osaka, 1-3 Yamadaoka, Suita, Osaka 565-0871, Japan; Graduate School of Frontier Biosciences, The University of Osaka, 1-3 Yamadaoka, Suita, Osaka 565-0871, Japan; JEOL YOKOGUSHI Research Alliance Laboratories, The University of Osaka, 1-3 Yamadaoka, Suita, Osaka 565-0871, Japan; JEOL Ltd., 3-1-2 Musashino, Akishima, Tokyo 196-8558, Japan; Department of Biophysics, Graduate School of Science, Kyoto University, Kitashirakawa Oiwake-cho, Sakyo-ku, Kyoto, Kyoto 606-8502, Japan; Department of Biophysics, Graduate School of Science, Kyoto University, Kitashirakawa Oiwake-cho, Sakyo-ku, Kyoto, Kyoto 606-8502, Japan; Department of Biophysics, Graduate School of Science, Kyoto University, Kitashirakawa Oiwake-cho, Sakyo-ku, Kyoto, Kyoto 606-8502, Japan; Department of Biophysics, Graduate School of Science, Kyoto University, Kitashirakawa Oiwake-cho, Sakyo-ku, Kyoto, Kyoto 606-8502, Japan; Department of Biophysics, Graduate School of Science, Kyoto University, Kitashirakawa Oiwake-cho, Sakyo-ku, Kyoto, Kyoto 606-8502, Japan; Institute for Protein Research, The University of Osaka, 3-2 Yamadaoka, Suita, Osaka 565-0871, Japan; Department of Complex Systems Science, Graduate School of Informatics, Nagoya University, Furo-cho, Chikusa-ward, Nagoya, Nagoya 464-8601, Japan; Institute for Protein Research, The University of Osaka, 3-2 Yamadaoka, Suita, Osaka 565-0871, Japan; Graduate School of Frontier Biosciences, The University of Osaka, 1-3 Yamadaoka, Suita, Osaka 565-0871, Japan; JEOL YOKOGUSHI Research Alliance Laboratories, The University of Osaka, 1-3 Yamadaoka, Suita, Osaka 565-0871, Japan; Department of Biophysics, Graduate School of Science, Kyoto University, Kitashirakawa Oiwake-cho, Sakyo-ku, Kyoto, Kyoto 606-8502, Japan; Department of Biophysics, Graduate School of Science, Kyoto University, Kitashirakawa Oiwake-cho, Sakyo-ku, Kyoto, Kyoto 606-8502, Japan

**Keywords:** amyloid fibrils, cryo-EM, neurodegenerative diseases

## Abstract

T-cell intracellular antigen-1 (TIA-1) is a key component of stress granules and contains an intrinsically disordered region called the prion-like domain (PLD). TIA-1 PLD can form condensates via phase separation that subsequently convert into amyloid fibrils. However, the structural basis underlying TIA-1 PLD amyloid fibril formation remains unclear, hindering clinical understanding and therapeutic intervention at the molecular level. Using cryo-electron microscopy, we identified structural features that are consistent with a labile architecture, including a kinked backbone conformation, a polar zipper, and a proline-mediated cross-β structure. We also determined the fibril structure containing a G355R missense mutation and found that this mutation disrupts the tight conformation around G355 in the wild-type structure, leading to delayed fibril formation. The amyloid fibril structures of TIA-1 PLD provide an atomic-level framework for mechanistic studies of mutation-driven dysfunction.

Significance Statement T-cell intracellular antigen-1 (TIA-1) is a multifaceted RNA-binding protein involved in stress granule formation, alternative splicing, apoptosis regulation, and mRNA translation. Missense mutations in its prion-like domain (PLD) have been linked to diverse neurodegenerative diseases, including amyotrophic lateral sclerosis, frontotemporal dementia, Welander distal myopathy, and multisystem proteinopathies. These mutations may reshape the conformational landscape of the PLD and modulate the condensate properties of TIA-1 and stress granules, yet the underlying molecular basis remains elusive. Here, we present cryo-electron microscopy structures of wild-type and G355R-mutant TIA-1 PLD amyloid fibrils, revealing how this single mutation alters fibril architecture and the kinetics of fibril formation. These atomic-level structures provide a platform for future studies exploring how PLD conformations govern TIA-1's diverse cellular functions and roles in diseases.

## Introduction

Amyloid fibrils are protein aggregates with an elongated, unbranched, and filamentous morphology that are 5 to 20 nanometers wide and several micrometers long. Their skeletal structure is composed of a cross-β structure in which the β-strands are arranged in a direction perpendicular to the fibril axis via backbone hydrogen bonds. Two cross-β structures often adopt a steric zipper interaction facing each other on the surface of the protruding side chains, which reinforces the structural stability of the amyloid fibrils (1–4). Amyloid fibrils were originally discovered as a component of proteinaceous deposits that accumulate inside and outside neurons, a pathological hallmark of neurodegenerative diseases such as Alzheimer's and Parkinson's diseases (5). It has been proposed that the misfolding of proteins such as amyloid-β and tau triggers the formation of amyloid fibrils that propagate to form macroscopic deposits (6). Amyloid fibrils involved in diseases are called pathogenic amyloids.

On the other hand, recent studies have shown that a variety of peptides and proteins in diverse species can also form amyloid fibrils (7). These molecules exploit amyloid fibril formation for peptide hormone secretion and bacterial survival strategies with biofilms (8, 9), suggesting that amyloid fibrils play physiological roles. Amyloid fibrils involved in physiology are called functional amyloids. Several studies have reported that some intrinsically disordered proteins (IDPs) can form phase-separated condensates via self-assembly properties and undergo a transition from condensates into amyloid fibrils (10–13). In such IDPs, amyloid fibril formation has been proposed to store mRNA molecules released by translation inhibition during stress responses (14), and to cause the switching of binding partners by presenting new molecular surfaces resulting from amyloid fibril formation, possibly involving gene regulation of transcription and translation (15, 16). The broad implications of amyloid fibril formation in various biological events, including its association with neurodegenerative diseases, indicate the need to discuss a detailed molecular mechanism based on amyloid fibril structures at the atomic level to better understand biological phenomena.

Amyloid fibril structures derived from self-assembly IDPs have been determined, such as the RNA-binding protein FUS, the RNA-processing protein hnRNPA2, and TDP-43, which is associated with amyotrophic lateral sclerosis (ALS) and frontotemporal lobar degeneration. These fibril cores are composed of short peptides of six to 10 residues or segments of several dozen residues derived from these IDPs (17–27). In their structures, structural features that ensure reversibility have been observed. These include, for example, kinked backbone conformations and extended β-strand motifs, which are suggested to partially destabilize intermolecular interactions (17, 19, 20, 22, 28, 29); aligned arrangements of charged amino acids, which generate electrostatic repulsion (27); and a polar zipper in which hydrophilic polar residues interlock with each other (21, 25, 30). These structural features, which are prevalent in amyloid fibrils, are implicated in conferring the instability inherent in functional amyloids and in the regulation of physiological functions established by their reversible self-assembly (4).

Recent advances in genetic analysis have enabled the identification of amino acid mutations associated with neurodegenerative diseases in several self-assembly IDPs (31). Many of these mutations have been found to accelerate the transition from condensates into amyloid fibrils (11, 13, 20, 29, 32, 33) and to reinforce the conformational stability of amyloid fibrils (22, 23, 27), raising the hypothesis that pathogenic amyloids may be a consequence of the transition process (12, 13, 31, 34). This model suggests that functional amyloids regulate physiological functions by reversibly assembling and disassembling, but disease-associated mutations induce aberrant conformational changes, resulting in the development of pathogenic amyloids with altered reversibility. However, it is not fully understood what kind of conformational changes the disease-associated mutations induce and how they modulate their reversibility, resulting in the transformation of functional amyloids into pathogenic amyloids.

T-cell intracellular antigen-1 (TIA-1), identified as a key component of stress granules (35), is reportedly involved in alternative splicing (36, 37), apoptosis regulation (38), and mRNA translation (39, 40). Furthermore, TIA-1 expression levels in the brain's hippocampus are implicated in synaptic plasticity and behavior in mice (41). Genetic analysis of neurodegenerative diseases such as ALS, frontotemporal dementia (FTD) (42), Welander distal myopathy (WDM) (43, 44), and multisystem proteinopathies (MSPs) (45, 46) has revealed that TIA-1 carries disease-associated mutations in its IDP region, the prion-like domain (PLD). The genetic causality of TIA-1 in ALS/FTD remains uncertain due to the absence of TIA-1 deposits in patients with TIA-1 mutations (42, 47), and inconsistent conclusions across different genetic analysis methods and datasets (48, 49). However, recent evidence revealed phenotypic variability of TIA-1 mutations: a two-generation family study showed the mother presenting WDM while her two daughters developed ALS (50), highlighting the complex pathogenic effects associated with the TIA-1 mutations. TIA-1 missense mutations have been shown to alter the self-assembly properties of liquid–liquid phase separation and subsequent amyloid fibril formation of TIA-1 (42, 51, 52). Given that the self-assembly mechanism underlies stress granule formation and possibly other physiological functions (35, 53, 54), these results suggest that structural studies of TIA-1 at the single amino acid residue level will contribute to future genetic analyses of TIA-1.

Pathological investigations across various neurodegenerative diseases have further illuminated TIA-1's role in protein aggregation. It has been demonstrated that TIA-1 co-aggregates with pathological proteins such as huntingtin and phosphorylated tau in brain tissue from mouse models of tauopathy and Huntington's disease, as well as in patients affected by Alzheimer's disease (55, 56). In vitro experiments show that TIA-1 has the ability to cross-seed with huntingtin and the yeast prion protein Sup35 to form two-protein amyloid fibrils (53, 55). These results imply that TIA-1 has the potential to serve as a platform for amyloid fibril formation. However, the structural basis of amyloid fibril formation at the atomic level has not been elucidated. In this study, we investigated the amyloid fibril structure formed by human wild-type (WT) TIA-1 PLD and found that amyloid fibrils have structural features that are consistent with a labile architecture, including a kinked backbone conformation, a polar zipper, and a proline-mediated cross-β motif. We also examined the amyloid fibril structure with a G355R missense mutation in the fibril core region, and found that G355R disrupts the tight conformation surrounding G355 in the WT fibril structure, resulting in delayed amyloid fibril formation. The amyloid fibril structures of TIA-1 PLD provide an atomic-level framework for mechanistic studies of mutation-driven dysfunction.

## Results

### Missense mutations in TIA-1 PLD alter the transition process from condensates to amyloid fibrils

**Fig. 1.**
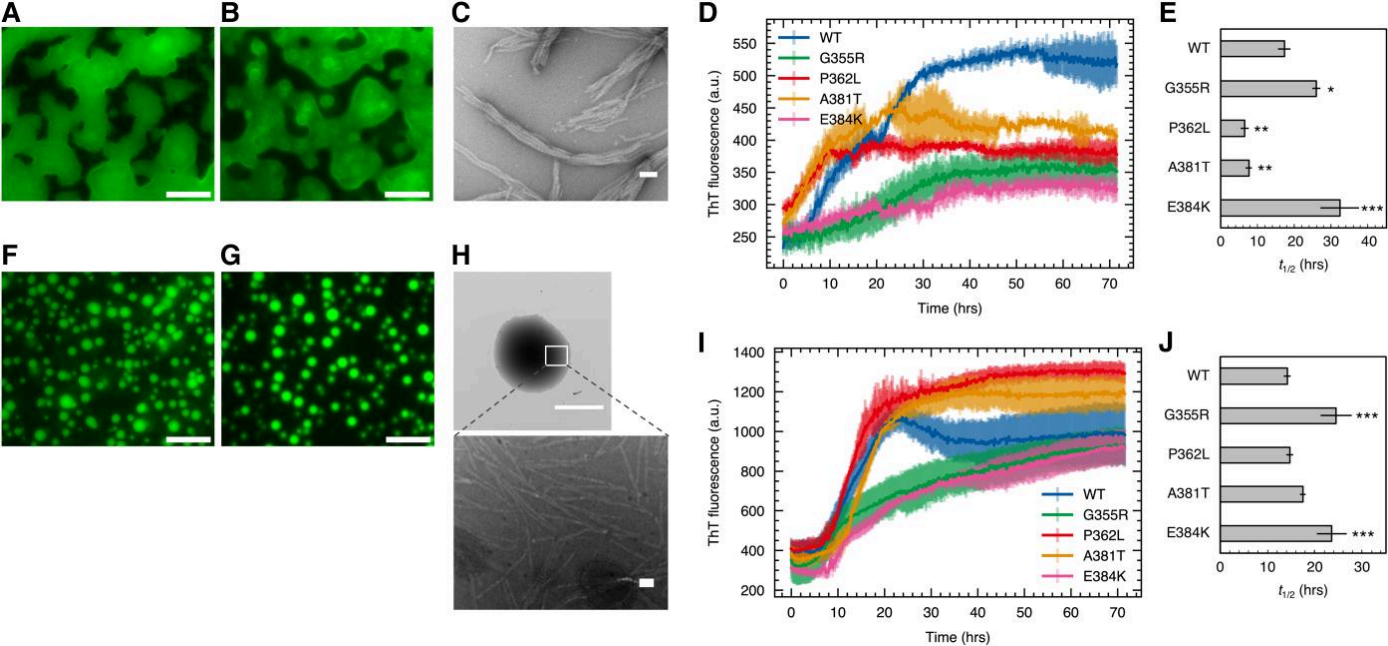
Missense mutations in TIA-1 PLD alter the transition process from condensates to amyloid fibrils. A and B) Fluorescence microscopy images of the full-length TIA-1 condensates before (A) and after incubation (B). Condensates were visualized by adding ThT. Under constant imaging parameters, ThT intensity within condensates remained visually unchanged over 72 h, suggesting that the signal originates from the high viscosity of the condensates. Scale bar, 10 μm. C) Negative staining TEM image of sample B. Scale bar, 100 nm. D and E) ThT assay (D) and *t*_1/2_ (E) of full-length TIA-1 fibril formation. Data are means ± SD. *n* = 2 biological replicates with three technical replicates each. *P < 0.05, **P < 0.01, ***P < 0.001 compared with WT by one-way ANOVA with a post hoc Tukey honest significant difference test. F and G) Fluorescence microscopy images of TIA-1 sPLD condensates before (F) and after incubation (G). Condensates were visualized by adding ThT. Scale bar, 10 μm. H) Negative staining TEM image of sample (G). The top panel shows a representative image of an aggregate, and the bottom panel shows an expanded view of the white rectangular region in the top panel. Scale bar, 5 μm for the top panel and 100 nm for the bottom panel. I and J) ThT assay (I) and *t*_1/2_ (J) of TIA-1 sPLD fibril formation. Data are means ± SD. *n* = 2 biological replicates with four technical replicates each. *P < 0.05, **P < 0.01, ***P < 0.001 compared with WT by one-way ANOVA with a post hoc Tukey honest significant difference test.

Full-length TIA-1, which contains three RNA recognition motifs (RRMs) followed by PLD, reportedly undergoes phase separation and subsequent amyloid fibril formation in the presence of nucleic acids (DNA or RNA) that specifically bind to tandem RRMs (57). In TIA-1 PLD, missense mutations, including P362L, A381T, and E384K, have been shown to influence the formation of phase-separated condensates and the process of SG disassembly (42, 51). Additionally, we focused on the G355R mutation in this study. This mutation was identified in the genetic research related to ALS/FTD (42), and our previous NMR analysis revealed an aromatic ring current shift of G355, indicating a distinct conformation with aromatic residues surrounding the residue. To investigate the consequences of these missense mutations on the transition process from condensates to amyloid fibrils, we analyzed the condensate properties of recombinant WTe and mutant TIA-1 proteins. The full-length human TIA-1 WT and its mutants (G355R, P362L, A381T, E384K) formed condensates generated via phase separation in the presence of single-stranded DNA TC5, which specifically binds to TIA-1 under physiological buffer conditions (Figs. 1A and S1). Fluorescence recovery after photobleaching (FRAP) analysis of the full-length WT condensates showed slow recovery, suggesting restricted molecular mobility within the condensates (Fig. S2A and B). After 3 days of incubation at 30 °C, although the condensates maintained their macroscopic morphology, the sample contained amyloid fibrils, as confirmed by negative staining transmission electron microscopy (TEM; Fig. 1B and C). To monitor the transition process in a time-dependent manner, we performed the thioflavin T (ThT) assay, a fluorescence assay to detect amyloid fibril formation. The results showed an increase in fluorescence intensity in WT and all mutants, but with differences in their formation rates (Fig. 1D). To compare the rate of amyloid fibril formation, we estimated *t*_1/2_, the time to reach half of the maximum fluorescence intensity, and found that P362L and A381T had a shorter *t*_1/2_ than WT, whereas G355R and E384K had a longer *t*_1/2_ (Fig. 1E).

We previously reported that the 320–386 residue region of mouse TIA-1 forms phase-separated condensates (51). Therefore, we investigated whether the same region of human TIA-1 (short PLD: sPLD) undergoes phase separation and subsequent amyloid fibril formation. Human TIA-1 sPLD formed condensates even in the absence of TC5 and FRAP analysis of the WT sPLD condensates showed very little fluorescence recovery, suggesting highly restricted molecular mobility within the condensates (Figs. 1F and S2C, D). After incubation at 30 °C for 3 days, TIA-1 sPLD condensates maintained their spherical morphology, whereas amyloid fibrils were observed within the condensates (Fig. 1G and H), indicating that TIA-1 sPLD condensates underwent a transition to amyloid fibrils. The mutant proteins formed condensates similar to WT, but there were differences in morphology. Specifically, P362L and A381T formed clump-like aggregates, while G355R and E384K retained their spherical morphology similar to WT (Fig. S3). The ThT assay of TIA-1 sPLD condensates showed that G355R and E384K had a longer *t*_1/2_ than WT, which corresponds to the results for the full-length TIA-1 (Fig. 1I and J). Additionally, negative staining TEM of the samples after the ThT assay showed long and intertwined fibrils similar to those observed for WT (Fig. S4). Collectively, these results demonstrate that missense mutations in TIA-1 PLD modulate both the properties of phase-separated condensates and the kinetics of their transition into amyloid fibrils, underscoring distinct mutation-specific effects on fibril formation.

### Amyloid fibril structure of WT TIA-1 sPLD

**Fig. 2.**
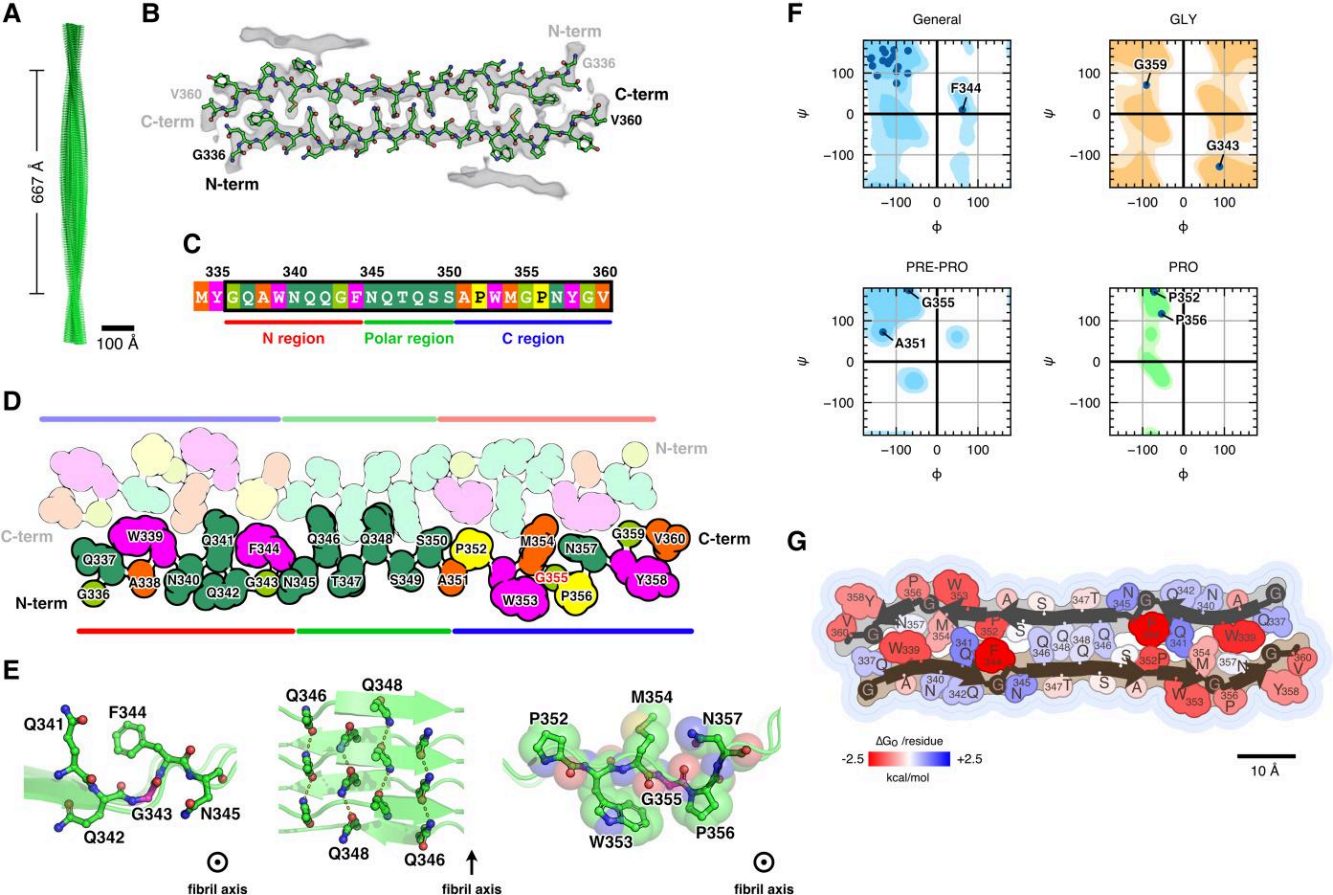
The amyloid fibril structure of WT TIA-1 sPLD. A) Overall view of the amyloid fibril structure of WT TIA-1 sPLD. B) A stick-and-ball model diagram of the WT fibril core structure. Density maps constructed by cryo-EM analysis are shown in gray. C) Amino acid sequence of WT TIA-1 sPLD. The region boxed in black is the core region of the WT TIA-1 sPLD fibril. Polar residues, hydrophobic residues, aromatic residues, glycine, and proline are shown in green, orange, magenta, yellow‒green, and yellow, respectively. The upper part shows the residue number of TIA-1, and the lower part shows the names of the three regions divided by structural features. D) A schematic diagram of the WT fibril core structure. The color scheme is the same as in C. One of the two protofilaments is shown in darker color, and the other is shown in lighter color. The lower part shows the names of the three regions divided by structural features. E) A stick-and-ball model diagram of the structural features in the WT fibril core structure. The semitransparent ribbon model diagram is superimposed. The left panel shows the kinked backbone conformation in the N region, the middle panel shows the polar zipper in the polar region, and the right panel shows the conformation around G355 with the semitransparent CPK (Corey–Pauling–Koltun) model. G343 and G355 are shown in magenta, and other residues are shown in green. The yellow dashed lines represent hydrogen bonds. The fibril axis is indicated in the lower right corner of each panel. The arrow indicates the vertical axis, and the circled dot indicates the depth axis of the fibrils. F) Ramachandran plots of residues forming the WT fibril core structure. Residues are divided into general residues (General), glycine (GLY), proline (PRO), and one residue before proline (PRE-PRO). Representative residues are labeled. G) Solvation free energy of the WT fibril core structure. Residues with positive values are colored blue, those with negative values are colored red, and a more negative value indicates a greater stabilizing contribution.

We performed cryo-electron microscopy (cryo-EM) analysis to elucidate the amyloid fibril structure formed by WT TIA-1 sPLD. A prerequisite for the cryo-EM analysis of amyloid fibrils is the production of long and dispersed fibrils in a specimen, but the amyloid fibrils of WT TIA-1 sPLD derived from condensates were not sufficiently dispersed for single particle analysis. Therefore, to improve fibril dispersion, we applied sonication to facilitate fibril transition and generate seeds, which were subsequently introduced into a monomer solution in 0.1% trifluoroacetic acid. The fibrils formed by the seeding method shared similar morphology with those formed by condensate maturation, featuring straight, unbranched structures ∼13–14 nm in width, while being more dispersed on the grid (Fig. S5). Cryo-EM analysis revealed two structural types of the amyloid fibrils formed by WT TIA-1 sPLD: flat fibrils and twisted fibrils. The proportions were 79.3% flat fibrils and 20.1% twisted fibrils (Fig. S6A). Although we could not solve the structure of flat fibrils due to insufficient data, we determined the structure of twisted fibrils at a resolution of 3.4 Å (Figs. 2A and S7A, B, Table 1). As discussed later, the amyloid fibrils of G355R TIA-1 sPLD predominantly have the twisted structure (Fig. S6B), allowing comparison of the same structural type in WT and G355R.

**Table 1.** Cryo-EM data collection and refinement statistics.

	TIA-1 WT fibrils	TIA-1 G355R fibrils
Data collection and processing		
Magnification	50,000	50,000
Voltage (kV)	300	300
Electron exposure (e−/Å^2^)	60	60
Defocus range (mm)	−1.0 to −2.5	−1.0 to −2.5
Pixel size (Å)	1.000	1.000
Symmetry imposed	C1	C1
Initial particle images (no.)	610,417	341,191
Final particle images (no.)	56,747	45,382
Map resolution (Å)	3.36	3.10
FSC threshold	0.143	0.143
Map resolution range (Å)	3.23 to 12.60	3.00 to 8.32
Refinement		
Initial model used (PDB code)	De novo	De novo
Model resolution (Å)	3.60	3.50
FSC threshold	0.5	0.5
Map sharpening *B* factor (Å^2^)	−48.16	−40.04
Model composition		
Nonhydrogen atoms	1,164	792
Protein residues	150	96
Ligands	0	0
*B* factors (Å^2^)		
Protein	75.78	90.3
Root-mean-square deviation		
Bond lengths (Å)	0.002	0.006
Bond angles (°)	0.487	0.929
Validation		
MolProbity score	1.96	2.22
Clashscore	12.5	18.52
Poor rotamers (%)	0	0
Ramachandran plot		
Favored (%)	94.93	92.86
Allowed (%)	5.07	7.14
Disallowed (%)	0	0

The amyloid fibril structure of WT TIA-1 sPLD consisted of two intertwined protofilaments (Fig. 2B), which together formed the cross-β spine. The β-strands of the two protofilaments adopted a helical arrangement with pseudo-2_1_ symmetry; each β-strand was displaced 2.38 Å along the fibril axis and rotated by 179.37° (Fig. S8A). Because of this symmetry, successive β-strands within a single protofilament were spaced 4.76 Å apart. The crossover distance of the fibril structure, defined as the distance over which a single β-strand rotates 180°, was 667 Å (Fig. 2A). This pseudo-2_1_ symmetry is also characteristic of other amyloid fibrils, including those formed by tau, α-synuclein, and Aβ42 (58–60). The WT fibrils adopt a linear conformation with TIA-1 sPLD residues 336–360 forming the fibril core (Fig. 2B and C), which corresponds to the region assigned as forming β-sheets of the TIA-1 PLD in the solid-state NMR analysis (52). The remaining regions, including the N- and C-termini, were not resolved in the density map, indicating structural flexibility or disorder. Notably, amino acid residues whose protonation states change under low pH conditions, such as Asp, Glu, and His, were absent in the fibril core, implying that the interaction modes observed in the fibril structure are unlikely to result from the low pH environment. Based on structural features, we divided the amyloid fibril structure into three parts: the N region (residues 336–344), the polar region (residues 345–350), and the C region (residues 351–360). The N region of one protofilament interacted with the C region of the other, and the polar region of one protofilament interacted with the same region of the other, so that the two β-sheets faced each other in a head-to-tail fashion (Fig. 2C and D).

In the N region, aromatic residues W339 and F344 interacted with P352 and M354 in the C region. The kinked backbone conformation formed by _341_QQGFN_345_ (Fig. 2E, left) is notable for disrupting the linearity of the fibril core, causing a shift in the β-strands of the two protofilaments. Ramachandran plots of the WT fibril structure revealed that G343 and F344 adopted conformations that deviated from the β-strand structure and resembled the conformations of low-complexity, aromatic-rich, kinked segments (LARKS; Fig. 2F). Given that LARKS have been proposed to contribute to the reversibility of IDP condensates by maintaining the solvated surface area of IDPs (4, 17, 28), the kinked backbone conformation centered on G343 may play a similar role in the reversibility of the WT fibrils.

The polar region is composed entirely of polar residues, such as glutamine and serine residues, and the side chains interlock with each other to form a steric zipper interaction, known as the polar zipper (Fig. 2E, middle). In the polar zipper, the glutamine side chains are aligned along the fibril axis to form a ladder of hydrogen bonds that stabilize the fibril structure, and their hydrophilicity is implied to contribute to the reversibility of functional amyloids (4), as observed in the amyloid fibrils of FUS (21, 25, 30). The solvation free energy estimated from the solvation surface area (4, 61) shows that the polar region has a lower energy than the N or C region, which contains several hydrophobic residues (Fig. 2G).

The C region consists of _351_APWMGPNYGV_360_, which contains two proline residues. Due to the imide bond in the backbone, prolines cannot form hydrogen bonds via an amide hydrogen. Therefore, they are not suitable for amyloid fibril formation with a cross-β structure. In fact, using a biochemical technique that converts an amide proton to a methyl group in a backbone, it has been shown that a proline in tau and hnRNPA2 inhibits amyloid fibril formation due to defects in hydrogen bonding via an amide hydrogen in a backbone (33). As shown above, the amyloid fibril structure of WT TIA-1 sPLD contains several structural features that are consistent with a labile architecture.

### Amyloid fibril structure of G355R TIA-1 sPLD

The amyloid fibril core of WT TIA-1 sPLD encompassed G355 at the G355R mutation site. As described previously, G355R exhibited a delay in amyloid fibril formation (Fig. 1D and I). G355 of the WT fibril was located in the C region and was confined to a tight conformation surrounded by W353 and P356 due to the compactness of the glycine residue (Fig. 2E, right). Therefore, we surmised that the G355R mutation disrupts the tight conformation of the C region present in the WT fibril structure through steric hindrance with surrounding residues.

**Fig. 3.**
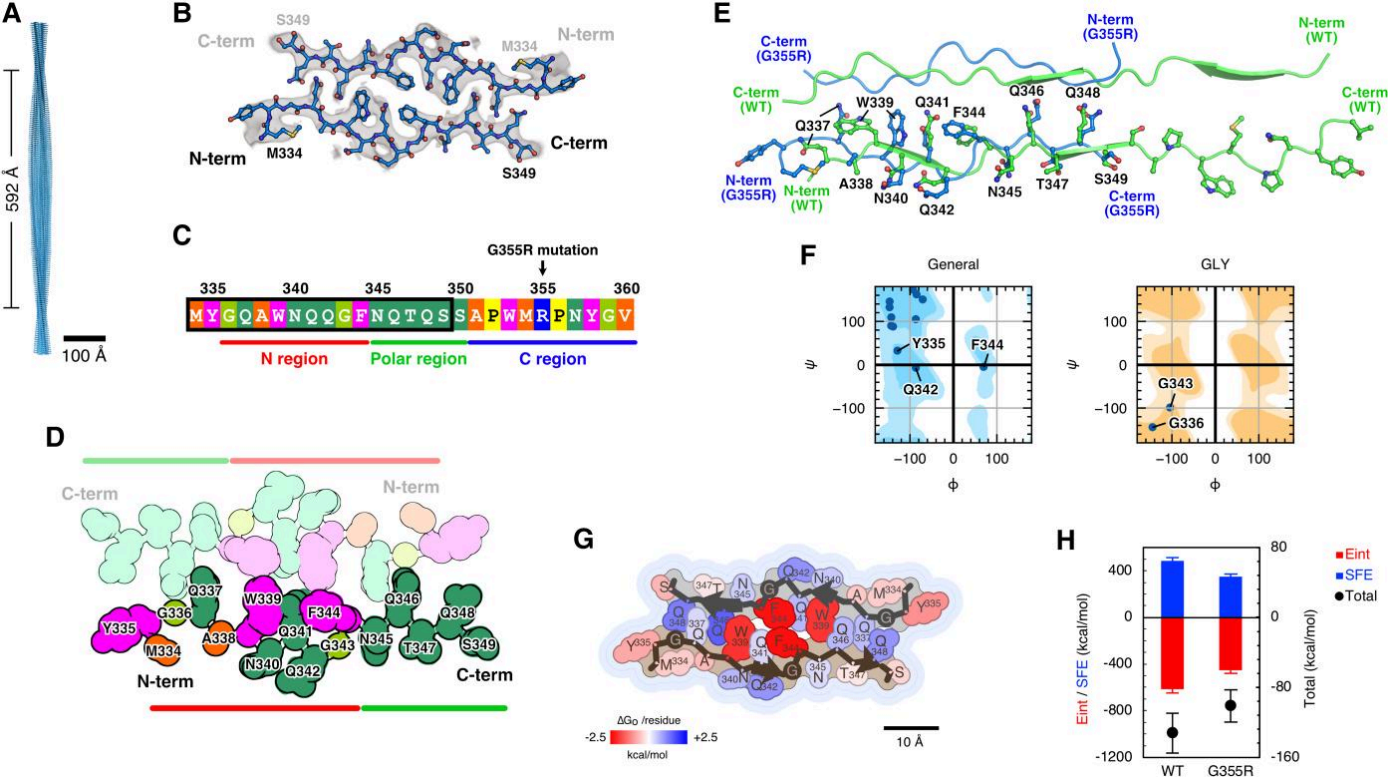
The amyloid fibril structure of G355R TIA-1 sPLD. A) Overall view of the amyloid fibril structure of G355R TIA-1 sPLD. B) A stick-and-ball model diagram of the WT fibril core structure. Density maps constructed by cryo-EM analysis are shown in gray. C) Amino acid sequence of G355R TIA-1 sPLD. The region boxed in black is the core region of the G355R TIA-1 sPLD fibril. The color scheme is the same as in WT. The upper part shows the residue number of TIA-1, and the lower part shows the names of the three regions divided by structural features. The G355R mutation site is indicated by an arrow. D) A schematic diagram of the G355R fibril core structure. The color scheme is the same as in C. One of the two protofilaments is shown in darker color, and the other is shown in lighter color. The lower part shows the names of the regions divided by structural features. E) A comparison of the WT and G355R fibril core structures. Backbone structures are represented by a semitransparent ribbon model diagram, and side chains are represented by stick-and-ball models. WT and G355R are shown in green and blue, respectively. The structures of the N and polar regions in one protofilament of WT and G355R are superimposed, and the N-terminus, C-terminus, and residues used for superposition are labeled. F) Ramachandran plot of residues forming the G355R fibril core structure. Residues are divided into general residues (General), glycine (GLY), proline (PRO), and one residue before proline (PRE-PRO). Representative residues are labeled. G) Solvation free energy (SFE) of the G355R fibril core structure. The color scheme is the same as in the WT. H) The stabilization energy required for the monomer to transfer into the fibril core calculated by MD/3D-RISM. The blue bar represents the SFE change for dehydration of the monomer to transfer into the fibril core, and the red bar represents the structural energy (Eint) of proteins, which includes both the conformational energy and the protein‒protein interaction energy. The total is the sum of SFE and Eint. MD simulations were performed for 100 ns, and 100 structures were collected every 1 ns for energy calculations. The bars represent the mean value of the SFE and Eint energies, and the error bars represent the SD.

To validate our assumptions, we performed cryo-EM analysis to elucidate the amyloid fibril structure formed by G355R TIA-1 sPLD. We used the same methods for cryo-EM sample preparation as for the WT fibrils. Cryo-EM analysis revealed that the G355R fibrils adopt one structural type of twisted fibrils (Fig. S6B). We determined the twisted fibril structure at a resolution of 3.1 Å (Figs. 3A and S7C, D, Table 1). The G355R fibril structure was composed of two intertwined protofilaments with a crossover distance of 592 Å, and the two protofilaments showed pseudo-2_1_ symmetry with an axial rise of 2.38 Å and a twist of 179.29° (Figs. 3A and S8B).

The amyloid fibril core of the G355R fibril structure was composed of residues 334–349, corresponding to the N and polar regions of the WT fibril structure (Fig. 3B–D), whereas the C region containing the G355R mutation site was not part of the amyloid fibril core. This result confirms the importance of the G355 residue for the formation of the C region, as suggested by the WT fibril structure. Since the G355R mutation replaces glycine with arginine with a larger side chain, we speculate that this mutation disrupted the tight conformation surrounding G355 in the WT structure, resulting in the loss of the interaction surface in the C region.

The structure of the N and polar regions of G355R was similar to that of WT with a root-mean-square distance of 2.495 Å for all atoms (Fig. 3E). It is noteworthy that, despite being derived from an IDP region with a random coil conformation in solution, these segments of WT and G355R adopt similar conformations in amyloid fibrils. On the other hand, their intermolecular interactions within the fibrils differ substantially between G355R and WT (Figs. 2D and 3D). We surmise that TIA-1 sPLD conserves interaction surfaces for amyloid fibril formation due to the bias of the conformational ensemble in the dynamic local structures, while their interaction modes have a degree of flexibility. Structural analysis of α-synuclein and tau fibrils has shown that fibril polymorphs can arise from differences in the interaction modes between core-forming building blocks, while the amino acid regions of the building blocks are conserved to some extent (4, 62). In TIA-1, the conserved interaction sites indicated by the WT and G355R TIA-1 sPLD fibril structures may contribute to fibril polymorphism.

Regarding the fibril core conformation, the Ramachandran plot of the G355R fibril structure shows that G343 and F344 adopt a LARKS conformation, as also observed in the WT fibril structure, and that G336 adopts a conformation similar to that of extended amyloid-like low-complexity segments (EAGLS), in which both backbone dihedral angles ϕ and ψ are in regions smaller than −150° (Fig. 3F). EAGLS is a structural feature prevalent in functional amyloids such as LARKS and has been suggested to contribute to weakened intermolecular interactions (28). These conformational features may influence the kinetics of fibril formation by modulating the strength and specificity of inter-residue contacts.

The fibril structures of WT and G355R differed in core length, conformational features, and interaction modes (Figs. 2G and 3G). To quantitatively evaluate the impact of these structural differences on the fibril formation of TIA-1 sPLD, we calculated the stabilization energy required for the monomer to transfer into the fibril core using MD/3D-RISM (Fig. 3H). The results showed that the dehydration free energy loss upon the transfer is compensated by the binding energy gain associated with fibril formation, resulting in negative values of stabilization energy in both WT and G355R. However, G355R exhibited a less favorable stabilization energy than WT due to the predominance of dehydration energy loss over binding energy gain. This energetic profile is consistent with the delayed fibril formation observed in G355R.

### The fibril core region is crucial for TIA-1 amyloid fibril formation

**Fig. 4.**
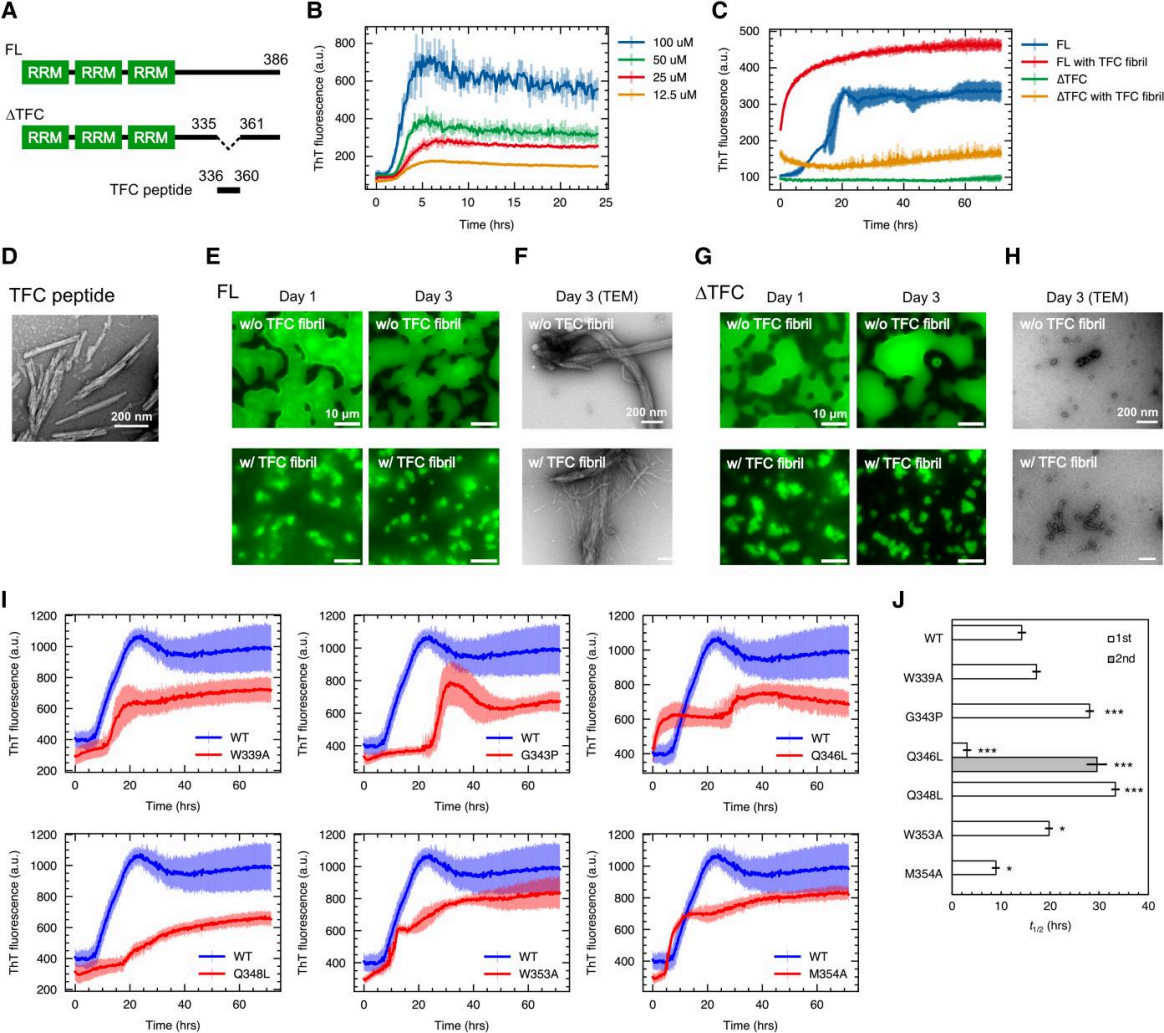
Missense mutations in the TIA-1 fibril core region affect the amyloid fibril formation. A) Domain architecture and the fibril core region of TIA-1. The full-length TIA-1 (FL) contains three RRM domains, followed by PLD. ΔTFC is a deletion mutant lacking the fibril core region spanning residues 336–360, and the TFC peptide is a synthetic peptide corresponding to the region. B) ThT assay of the TFC peptide. Data are the mean ± SD. *n* = 2 biological replicates with four technical replicates each. C) ThT assay of FL and ΔTFC TIA-1 and the seeding effects of the TFC peptide fibrils. Data are the mean ± SD. *n* = 2 biological replicates with four technical replicates each. D) Negative staining TEM image of the TFC peptide fibrils. Scale bar, 200 nm. E and G) Fluorescence microscopic images of FL (E) and ΔTFC (G) TIA-1 condensates with or without the TFC peptide fibrils. Condensates were visualized with the addition of ThT, and the fluorescence images were acquired at day 0 and after 3 days of incubation. Scale bar, 10 μm. F and H) Negative staining TEM images of FL (F) and ΔTFC (H) TIA-1 condensates with or without the TFC peptide fibrils. The TEM images were acquired after 3 days of incubation. Scale bar, 200 nm. I) ThT assay of TIA-1 sPLD fibril formation for WT and mutants. Each panel shows a comparison of WT and each mutant, with WT shown in blue and a mutant shown in red. Data are the mean ± SD. *n* = 2 biological replicates with four technical replicates each. J) *t*_1/2_ of the TIA-1 sPLD fibrils for WT and mutants. Regarding Q346L, a *t*_1/2_ from 0 to 30 h is shown as first, and *t*_1/2_ from 30 to 72 h as second. Data are the mean ± SD. *n* = 2 biological replicates with four technical replicates each. *P < 0.05, **P < 0.01, ***P < 0.001 compared with WT by one-way ANOVA with a post hoc Tukey honest significant difference test.

The core region of the WT TIA-1 sPLD fibril consisted of only 25 amino acid residues out of 386 residues in TIA-1 (Fig. 4A). We ascertained the role of the fibril core region in TIA-1 amyloid fibril formation. First, we conducted a ThT assay using a synthesized peptide of the 25-residue TIA-1 fibril core (TFC peptide). The TFC peptide exhibited rapid amyloid fibril formation in the concentration range of 100 to 12.5 μM, with a *t*_1/2_ of 4 h at 25 μM (Fig. 4B and D).

We also generated a recombinant protein of a TIA-1 deletion mutant lacking the fibril core region (ΔTFC; Fig. 4A). ΔTFC formed phase-separated condensates similar to full-length TIA-1 (Fig. 4E and G) but did not transition to amyloid fibrils based on the ThT assay and TEM analysis (Fig. 4C, F, and H). Interestingly, addition of preformed TFC peptide fibrils significantly accelerated amyloid fibril formation by full-length TIA-1, reducing the *t*_1/2_ from 16.9 ± 1.6 h without the TFC fibrils to 3.61 ± 0.1 h with the TFC fibrils, indicating a seeding effect. However, ΔTFC did not exhibit the seeding effect in the presence of the TFC peptide fibrils (Fig. 4C, F, and H). These findings suggest that the TIA-1 fibril core region is sufficient to drive rapid amyloid fibril formation, and necessary for full-length TIA-1 to transition from condensates to fibrils.

To investigate the correlation between the structural features revealed by the WT TIA-1 sPLD amyloid fibril structure and the transition process from condensates to amyloid fibrils, we performed a mutational analysis. The mutants we generated are W339A and M354A, in which a hydrophobic residue located at the interaction surface between the two protofilaments in the WT fibrils is mutated to alanine; G343P, in which the glycine located at the kinked backbone conformation is mutated to proline; Q346L and Q348L, in which a glutamine involved in the polar zipper is mutated to leucine; and W353A, in which a tryptophan located on the opposite side of the interaction surface between the protofilaments is mutated to alanine. For W353A, although polypeptide chain densities were observed around the W353 residues in the WT fibril structure, we were unable to construct the atomic model due to the lack of linkage to the fibril core (Fig. 2B). Therefore, we speculated that W353 may also be involved in amyloid fibril formation.

All mutants formed phase-separated condensates under physiological buffer conditions and retained their spherical morphology after 3 days of incubation (Fig. S9). We monitored the transition process from condensates to amyloid fibrils by the ThT assay and estimated *t*_1/2_ for the growth rate of each (Fig. 4I and J). For the mutation of hydrophobic residues located at the interaction interface, W339A showed a *t*_1/2_ similar to that of WT, and M354A had a slightly shorter *t*_1/2_ than WT. G343P, located in the kinked backbone conformation, showed a longer *t*_1/2_ as well as a late onset of fluorescence intensity increase compared with WT, suggesting a delay in the nucleation of amyloid fibril formation. In the polar region mutation Q346L, while WT showed a growth curve close to a sigmoid, Q346L showed a growth curve with two rapidly increasing phases. This result implies the formation of two types of fibrils with different formation rates by Q346L. Another mutation in the polar region, Q348L, also showed a longer *t*_1/2_ than WT, suggesting that the glutamine residues in the polar region are closely involved in amyloid fibril formation of TIA-1 sPLD. Finally, W353A, which is located on the opposite side of the interaction interface between the protofilaments, showed a slightly longer *t*_1/2_ than WT. In addition, TEM observation of the resulting fibrils revealed that all mutants formed fibrils with morphologies similar to that of WT (Fig. S10). These results suggest that structural features such as a kinked backbone conformation and a polar zipper in the WT fibril structure control the transition process from condensates to amyloid fibrils.

## Discussion

In this study, we conducted cryo-EM analysis of TIA-1 sPLD amyloid fibrils and observed that the fibril core region spanned residues 336–360. This region is highly prone to amyloid fibril formation and shows a seeding effect on full-length TIA-1. In TIA-1 sPLD, missense mutations associated with WDM, MSP, ALS, and FTD have been identified, and these mutations are located in and around the fibril core region. Among the missense mutations, P362L and A381T facilitated amyloid fibril formation, while G355R and E384K delayed it. The fibril core region of TIA-1 did not cover the mutation sites P362L, A381T, and E384K, but included the G355R mutation site. We analyzed the amyloid fibril structure of G355R and observed a reduction in its fibril core region compared with WT. Given that G355 is confined to a tight conformation in the WT fibril structure, we suggest that the conformational changes caused by the G355R mutation prevent it from maintaining the WT conformation. On the other hand, P362L and A381T, which were located outside of the fibril core region, facilitated amyloid fibril formation. We previously reported that these mutations augment intermolecular interactions by modulating local dynamics around the mutation sites (51). Given the results, we hypothesize that P362L and A381T may expand the region involved in fibril formation in addition to the fibril core region of WT. As for E384K, which delayed amyloid fibril formation, its mechanism remains unclear and warrants future investigation. The amyloid fibril structures of WT and G355R TIA-1 sPLD provide mechanistic insights into the transition process from condensates to amyloid fibrils and the kinetics of fibril formation induced by missense mutations.

Notably, our cryo-EM analysis of WT TIA-1 sPLD revealed two fibril morphologies—flat (∼80%) and twisted (∼20%)—although this ratio may reflect nonphysiological in vitro conditions. Because the flat fibrils lack helical symmetry, a high-resolution structure could be determined only for the twisted fibrils. The absence of flat fibril structures prevents us from assessing the biological and pathological significance of each polymorph. Untwisted flat polymorphs have been reported for other amyloid-forming proteins, including tau, human islet amyloid polypeptide, and α-synuclein (63–65). Recent time-resolved cryo-EM studies of IAPP-S20G and tau have suggested that untwisted morphologies can serve as on-pathway intermediates that eventually convert into mature twisted fibrils (63, 64), raising the possibility that the flat fibrils observed in WT TIA-1 sPLD represent an early stage of the condensate-to-fibril transition. Given that structural studies of ex vivo amyloid fibrils suggest that their polymorphism is disease specific (4), these findings underscore the importance of clarifying how these fibril polymorphs arise in cells and understanding their biological and pathogenic roles.

Cryo-EM analysis of WT TIA-1 sPLD uncovered structural features of the amyloid fibrils such as a kinked backbone conformation, a polar zipper, and a proline-mediated cross-β structure. These structural features are frequently observed in functional amyloids and are suggested to play a crucial role in their reversibility (4). Notably, within the TIA-1 fibril core region, two glycine residues (G343 and G355) adopted a kinked conformation, and mutations such as G343P and G355R have an impact on the kinetics of amyloid fibril formation. The amino acid sequence of self-assembly IDPs has been reported to be glycine rich (66). Glycine acts as a linker to maintain the flexible conformation of the IDPs within the condensates (67, 68) and is abundant in LARKS and EAGLS, which are the characteristic backbone conformations found in functional amyloids (17, 22), suggesting that glycine is required for the reversible self-assembly of IDPs. Indeed, in a genome-wide search for disease-associated mutations that induce a LARKS-like kinked conformation to a steric zipper-like pleated β-sheet conformation, many cases of glycine mutation to polar or hydrophobic residues have been identified (29). These results suggest that glycine plays an important role in the TIA-1 transition process from condensates to amyloid fibrils.

**Fig. 5.**
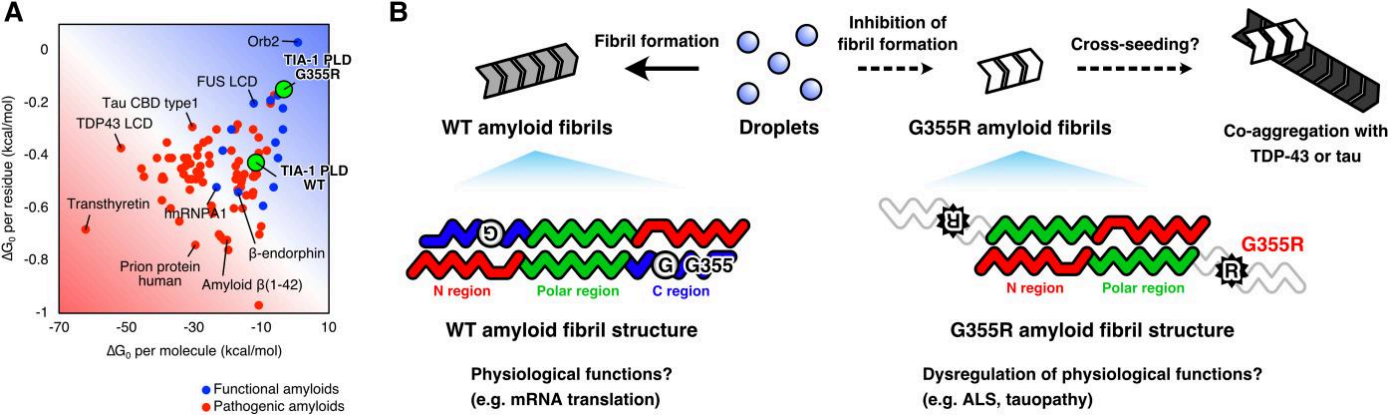
G355R mutation disrupts amyloid fibril formation through conformational changes. A) The solvation free energies of functional amyloids, pathogenic amyloids, and TIA-1 amyloid fibrils are plotted on a 2D map. The horizontal axis represents the solvation free energy per molecule, and the vertical axis represents the solvation free energy per residue. Solvation free energies are calculated using the solvation surface area estimated from amyloid fibril structures. Functional amyloids are shown in blue, pathogenic amyloids are shown in red, and WT and G355R TIA-1 sPLD amyloid fibrils are shown in green. Representative proteins are labeled. B) A schematic illustration of the inhibition mechanism of amyloid fibril formation by the G355R mutation. TIA-1 proteins undergo a transition from condensates into amyloid fibrils, but the ALS-associated G355R mutation induces conformational changes in the C region of the WT TIA-1 sPLD fibril structure, inhibiting amyloid fibril formation. This perturbation of fibril formation may cause dysregulation of physiological functions attributable to the WT fibrils, leading to ALS pathogenesis. Alternatively, it may enhance cross-seeding ability and promote co-aggregation with TDP-43 and tau.

Based on the amyloid fibril structures elucidated to date and by looking at the solvation free energy of amyloid fibrils in the big picture, it has been proposed that functional amyloids have a less stable fibril structure with a small negative value of the solvation free energy, while pathogenic amyloids have a more stable fibril structure with a large negative value of the solvation free energy (4) (Fig. 5A). The solvation free energies of the WT and G355R amyloid fibril structures estimated from the solvation surface area were −11.3 kcal/mol per molecule and −0.43 kcal/mol per residue for WT, while they were −3.1 kcal/mol per molecule and −0.15 kcal/mol per residue for G355R. Comparison of the solvation free energies of the WT and G355R amyloid fibrils with other amyloid fibrils shows that they are both located approximately in the functional amyloid region in a solvation free energy map and that the G355R fibril core is less stable than the WT core (Fig. 5A).

In general, amino acid mutations implicated in neurodegenerative diseases often enhance the structural stability of amyloid fibrils. However, the G355R mutation was found to inhibit amyloid fibril formation. This result suggests that the G355R mutation may impair physiological functions mediated by TIA-1 amyloid fibrils. TIA-1, similar to FUS and Orb2 (15, 16), potentially regulates physiological processes such as transcription and translation through amyloid fibril formation. We hypothesize that the G355R mutation disrupts the formation of functional amyloids, thereby impairing the intrinsic function of TIA-1 and contributing to disease pathogenesis through a loss-of-function mechanism (Fig. 5B). Indeed, structural studies of the ribonucleoprotein hnRNPDL-2 have demonstrated the potential role of loss-of-function mechanisms in disease development (69). It has been shown that hnRNPDL-2 can form amyloid fibrils while retaining its ability to bind oligonucleotides and that the missense mutation D259H/N associated with limb-girdle muscular dystrophy D3 suppresses amyloid fibril formation. These findings indicate that hnRNPDL-2 amyloid fibrils play a physiological role and that inhibiting their formation may lead to loss-of-function disease. The structural similarity between the WT TIA-1 sPLD fibril structure and functional amyloid suggests the potential of being functional amyloid. However, biological validation is essential to confirm the pathogenicity of G355R through loss-of-function mechanisms.

Another possibility is that the G355R TIA-1 fibrils participate in cross-seeding to induce amyloid fibril formation of other proteins, such as TDP-43 and tau. TIA-1 is known to form pathological co-aggregates with huntingtin and phosphorylated tau in vivo (55, 56) and to induce cross-seeding of huntingtin and yeast prion Sup35 in vitro (53, 55). These findings suggest that TIA-1 functions as an aggregation platform for other proteins. The conformational changes in amyloid fibrils induced by the G355R mutation may thus enhance their ability to cross-seed pathogenic amyloid formation with other proteins (Fig. 5B). Notably, pathological TDP-43 aggregates, which are closely associated with ALS pathology, were detected in autopsy samples from ALS patients carrying TIA-1 mutations, while TIA-1 aggregates were not observed in the same tissues (42, 47). However, this inability to detect TIA-1 aggregates may reflect technical limitations, such as epitope masking in the immunohistochemical analysis. Further investigation is needed to determine the relationship between TIA-1 amyloid fibril formation and ALS and FTD pathology.

In this study, we investigated how TIA-1 PLD missense mutations alter the transition process from condensates to amyloid fibrils. Previous studies have shown that these missense mutations affect not only amyloid fibril formation but also formation of phase-separated condensates (42, 51). Future studies will need to consider these molecular effects in the context of biological condensates to fully understand their impact on cellular functions. The atomic-level structural analysis of WT and G355R TIA-1 sPLD amyloid fibrils revealed that the G355R mutation disrupts the tight conformation surrounding G355 in the WT fibril structure, resulting in delayed amyloid fibril formation. Our cryo-EM structures of the TIA-1 PLD map mutation sites that could modulate molecular interactions, thereby providing testable hypotheses for future work. Integrating these atomic-level structures with in-cell and clinical investigations will help clarify how the PLD and its disease-associated mutations influence TIA-1-mediated cellular functions and pathogenesis.

## Materials and methods

### Cryo-EM measurements

An aliquot of 2 μL of WT or G355R TIA-1 sPLD fibrils was applied to glow-discharged (JEOL, JEC-3000 FC, 20 s) quantifoil holey carbon-supported copper grids (R 1.2/1.3, 300 mesh), blotted (Whatman #1) and plunge-frozen in liquid ethane using an EM GP (Leica) at 4 °C and 95% humidity. Image acquisition was performed on a CRYO ARM 300 (JEOL) system equipped with a cold field-emission gun and operated at 300 kV in bright field imaging mode, an Ω-type energy filter with a 20-eV slit width, and a K3 direct electron detector camera (Gatan). Data were collected using Serial-EM (70), and carbon holes were detected using YoneoLocr (71). Movies were recorded using a K3 detector (Gatan) in counting and CDS mode with hard binning at a nominal magnification of 50,000× at the camera level, corresponding to a pixel size of 1.00 Å with 60 frames at a dose of 1.00 e^−^/Å^2^ per frame and an exposure time of 5.353 s per movie resulting in a total dose of 60.0 e^−^/Å^2^. A total of 4,650 movies for WT or 6,050 movies for G355R were collected in series within a defocus range of 1.0 to 2.5 μm.

## Supplementary Material

pgaf388_Supplementary_Data

## Data Availability

The atomic coordinates and structure factors have been deposited in the Protein Data Bank, https://www.rcsb.org (PDB/EMDB ID codes: 9KTY/EMD-62570 for WT and 9KTZ/EMD-62571 for G355R). Complete experimental procedures can be found in Supplementary Materials and Methods in SI Appendix.

## Preprints

This manuscript was posted on a preprint at https://www.researchsquare.com/article/rs-2950744/v1?.
