# Supplementary material for: Structural basis for T-cell intracellular antigen-1 amyloid fibril formation revealed by cryo-electron microscopy
Source: PNAS Nexus. 2025 Dec 11;4(12):pgaf388. doi: 10.1093/pnasnexus/pgaf388 (PMC12723238; doi:10.1093/pnasnexus/pgaf388)
Supplement: pgaf388_Supplementary_Data [file pgaf388_supplementary_data.pdf]

Supporting Information for

Structural basis for TIA-1 amyloid fibril formation revealed by cryo-EM

Daigo Inaoka, Tomoko Miyata, Fumiaki Makino, Yasuko Ohtani, Miu Ekari, Ryoga Kobayashi, Kayo Imamura, Emi Sakamoto, Takashi S. Kodama, Norio Yoshida, Takayuki Kato, Keiichi Namba, Hidehito Tochio, Naotaka Sekiyama

Corresponding author

Naotaka Sekiyama (sekiyama.naotaka.5x@kyoto-u.ac.jp)

This PDF file includes:

Supporting text

SI References

Supplementary Figure 1 to 10

Supporting text

Materials and Methods

Plasmids for *E. coli* expression of full-length TIA-1

The DNA fragment encoding full-length TIA-1 was synthesized by GENEWIZ and subcloned into a pET28a/His10-SUMO3 vector. The expression construct encodes the following amino acid sequence.

MGSSHHHHHHHHHHSSGLVPRGSHMNNDHINLKVAGQDGSVVQFKIKRHTPLSKLMK
AYCERQGLSMRQIRFRFDGQPINETDTPAQLEMEDEDTIDVFQQQTGGSMEDEMPKTL
YVGNLSRDVTEALILQLFSQIGPCKNCKMIMDTAGNDPYCFVEFHEHRHAAAALAAM
NGRKIMGKEVKVNWATTPSSQKKDTSSSTVVSTQRSQDHFHVFVGDLSPEITTEDIKAA
FAPFGRISDARVVKDMATGKSKGYGFVSFFNKWDAENAIQQMGGQWLGGRQIRTNW
ATRKPPAPKSTYESNTKQLSYDEVVNQSSPSNCTVYCGGVTSGLTEQLMRQTFSPFGQI
MEIRVFPDKGYSFVRFNSHESAAHAIVSVNGTTIEGHVVKCYWGKETLDMINPVQQQN
QIGYPQPYGQWGQWYGNAQQIGQYMPNGWQVPAYGMYGQAWNQQGFNQTQSSAP
WMGPNYGVQPPQGQNGSMLPNQPSGYRVAGYETQ*

The plasmids G355R, P362L, A381T, and E384K were produced using the QuickChange method using the pET28a/His10-SUMO3-TIA-1 vector as a template.

Plasmids for *E. coli* expression of TIA-1 sPLD

The DNA fragment encoding TIA-1 sPLD (320-386) with His12 and Tobacco Etch Virus (TEV) protease site was synthesized at GENEWIZ and subcloned into a pET28a vector. The amino acid sequence for the expression is as follows.

MGSSHHHHHHHHHHHHSENLYFQGGQYMPNGWQVPAYGMYGQAWNQQGFNQTQS SAPWMGPNYGVQPPQGQNGSMLPNQPSGYRVAGYETQ*

The plasmids G355R, P362L, A381T, E384K, W339A, G343P, Q346L, Q348L, W353A, and M354A were produced by a quickchange method using the pET28a/His12-TEV-TIA-1 sPLD vector as a template.

Purification of full-length TIA-1

E. coli (BL21) cells were transformed with a plasmid of interest and cultured in Luria broth (LB); protein expression was induced with 0.1 mM isopropyl-β-D-1-thiogalactopyranoside (IPTG) when the OD_{600} value was greater than 1.0, and cells were cultured at 18˚C for 16 hours. Cells were harvested and resuspended in Tris buffer (50 mM Tris-HCl, pH 7.5, 300 mM NaCl, 5 mM 2-mercaptoethanol). To the cell suspension, 1 mM phenylmethylsulfonyl fluoride (PMSF) and 0.1% Triton-X were added, and the cells were lysed by sonication on ice. The cell

lysate was centrifuged at 15,000 rpm for 40 minutes and the supernatant was removed. Denaturing buffer (50 mM Tris-HCl, pH 7.5, 2 M guanidine hydrochloride (GdnHCl)) was added to the precipitate and the precipitate was dissolved in a homogenizer. The lysate was centrifuged again at 15,000 rpm for 20 minutes, and the supernatant was applied to a Ni-NTA column and incubated at room temperature for 2 hours. The resin was then washed with denaturing buffer, wash buffer (50 mM Tris-HCl, pH 7.5, 5 mM imidazole, 2 M GdnHCl), and finally eluted the protein of interest with elution buffer (50 mM Tris-HCl, pH 7.5, 250 mM imidazole, 2 M GdnHCl).

The eluate was concentrated to 5 ml by centrifugal ultrafiltration, and the concentrated sample was slowly added dropwise to 40 ml dilution buffer (20 mM Tris-HCl, pH 8.0, 300 mM NaCl, 400 mM arginine hydrochloride (ArgHCl), 20 mM DTT) for protein refolding. The concentrated sample was treated with the sentrin-specific protease (SENP) and incubated overnight at room temperature to cleave the His-SUMO tag. The samples were subjected to gel filtration on a HiLoad 16/600 Superdex 75 pg column (Cytiva) using buffer containing 50 mM Tris-HCl, pH 7.5, 60 mM KCl, 1 mM $MgCl_2$, 2 mM DTT, 0.5 M ArgHCl, 0.5 mM EDTA, and the fraction containing the protein was concentrated to 500 μM and stored at −80˚C. This protocol follows the method of Loughlin et al (1).

Purification of TIA-1 sPLD

The purification procedure was basically the same as for full-length TIA-1 until purification by Ni-NTA, but in the case of TIA-1 sPLD, 8 M urea was used as the denaturing agent instead of GdnHCl. The eluate from a Ni-NTA column was concentrated and dialyzed against 0.1% trifluoroacetic acid (TFA) solution to remove urea and then lyophilized. The resulting protein powder was dissolved in dimethyl sulfoxide (DMSO) to 3 mM and stored at –80 °C.

Condensate Sample Preparation and Fluorescence Microscopy

For full-length TIA-1, a stock sample of full-length TIA-1 was incubated at room temperature for 30 minutes. The synthetic DNA oligonucleotide TC5 (5’-TTTTTACTCCAATTTTTACTCCAATTTTTACTCCAATTTTTACTCCAATTTTTACTCC-3’) was synthesized and HPLC purified commercially (Eurofins). TC5 was diluted to 0.5 μM in fibrillation buffer (50 mM HEPES, pH 7.5, 150 mM NaCl, 1 mM DTT), then full-length TIA-1 was added to reach 10 μM. The sample was then incubated at room temperature for 30 minutes and used as a condensate sample.

For TIA-1 sPLD, a stock sample of TIA-1 sPLD was heated at 95 ˚C for 10 minutes and cooled to room temperature for 20 minutes. TIA-1 sPLD was diluted to 25 μM in the fibrillation

buffer, heated again at 95 ˚C for 10 minutes, and cooled to room temperature for 30 minutes and used as a condensate sample. Heating and cooling cycles were applied to allow for the reproducible formation of uniformly spherical condensates. In preliminary experiments, samples without this step contained a mixture of spherical and irregularly shaped condensates. This step served as a reset point to ensure the complete dissolution of invisible condensates and oligomers.

Fluorescence microscopy of the condensate samples was performed using an Olympus IX71 microscope equipped with a Hamamatsu ORCA-Flash4.0 V3 CMOS camera and a UPLXAPO100XOPH 100x/1.45 oil immersion objective lens. Condensates were visualized with the addition of 10 μM Thioflavin T (ThT) immediately before observation. A 100 μL condensate sample was placed on a glass-bottom dish, and ThT fluorescence was observed with the Olympus U-NIBA filter (excitation 470-495 nm, emission 510-550 nm) of the microscope.

Fluorescence recovery after photobleaching (FRAP)

Condensate samples of full-length WT and WT sPLD TIA-1 were prepared in HEPES buffer using the same protocol as described above. For FRAP analysis, ATTO488-labeled mouse TIA-

1 sPLD (labeling rate: 3.4%), previously used in our prior study (2), was added to the condensate samples at a final concentration of 1 μM.

FRAP measurements were performed at room temperature using a Zeiss LSM780 confocal microscope. ATTO488 fluorescence was acquired at 543 nm with excitation at 488 nm. Photobleaching was carried out using a 488 nm laser to produce a circular bleaching region. The fluorescence intensity was normalized such that the pre-bleaching level was set to 1.0 and the immediate post-bleaching level to 0. Recovery was recorded at 1.94-second intervals.

Thioflavin T (ThT) Assay and $t_{1/2}$ Estimation

Samples for the ThT assay were prepared in a 96-well black plate with 100 μl of the condensate sample as described above, supplemented with 10 μM ThT. ThT fluorescence measurements were performed at 30˚C for 72 hours and ThT fluorescence was monitored with excitation at 425 nm and emission at 485 nm using a SpectraMax M2 (Molecular Devices). Fluorescence measurements were performed at 10-minute intervals, and the samples were vibrated for 5 seconds prior to each measurement. All samples were examined in triplicate or quadruplicate. Buffer samples supplemented with 10 μM ThT were also measured, and the mean and standard

deviation of each sample were corrected using those of the buffer (i.e. $MV(corrected) = MV(sample) - MV(buffer),\ SD(corrected) = \sqrt{SD(sample)^2 + SD(buffer)^2}$).

The $t_{1/2}$ was calculated as the time to reach the mean of the 0 h value and the maximum value in the ThT assay plot for each sample, and the mean and standard deviation for all samples were plotted on a graph.

One-way ANOVA followed by a post hoc Tukey honest significant difference test was performed to compare the wild-type and mutants at the following website (https://www.statskingdom.com/180Anova1way.html).

Transmission Electron Microscopy (TEM) with Negative Staining

An 8 µl aliquot of the fibril samples or the sample after the ThT assay was applied to glow-discharged continuous carbon film-coated copper grids (Nisshin EM) and stained with a 2% (w/w) uranyl acetate solution. After brief blotting, the grids were air-dried and examined on a HITACHI H-7650 electron microscope at 80 kV. The magnification ranged from 15,000× to 20,000×. Scale bars in each figure were standardized to accurately reflect spatial dimensions.

Preparation of WT or G355R TIA-1 sPLD Fibrils for Cryo-EM

Condensate samples of WT and G355R TIA-1 sPLD were prepared in MES buffer (50 mM MES, pH 6.5, 150 mM NaCl, 1 mM DTT) using the same method as previously described. To produce fibril seeds, the condensate sample was then sonicated for 1 hour on ice using a Bioruptor UCD-250 ultrasonicator (Cosmo Bio) in cycles of 10 seconds on and 10 seconds off at high power. This sonication process facilitates the transition from condensates to fibrils and produces short fibril fragments that act as seeds. In this stage, heating and cooling cycles were not applied. A stock sample of TIA-1 sPLD was diluted to 100 μM in 0.1% trifluoroacetic acid (TFA), and the fibril seeds were added at 5%. The resulting sample was incubated at room temperature for at least one week and used for cryo-EM observation.

Cryo-EM measurements

An aliquot of 2 μl of WT or G355R TIA-1 sPLD fibrils was applied to glow-discharged (JEC-3000 FC, 20 s) quantifoil holey carbon-supported copper grids (R 1.2/1.3, 300 mesh), blotted (Whatman #1) and plunge-frozen in liquid ethane using a Leica EM GP at 4 ˚C and 95% humidity. Image acquisition was performed on a CRYO ARM300 (JEOL) system equipped with a cold field-emission gun and operated at 300 kV in bright field imaging mode, an Ω-type

energy filter with a 20-eV slit width, and a K3 direct electron detector camera (Gatan, USA). Images were recorded using Serial-EM (3) and carbon holes were detected using YoneoLocr (4). Movies were recorded using a K3 detector (Gatan) in counting and CDS mode with hard binning at a nominal magnification of 50,000× at the camera level, corresponding to a pixel size of 1.00 Å with 60 frames at a dose of 1.00 $e^{-}/Å^2$ per frame and an exposure time of 5.353 s per movie resulting in a total dose of 60.0 $e^{-}/Å^2$. A total of 4650 movies for WT or 6050 movies for G355R were collected in series within a defocus range of 1.0 μm to 2.5 μm.

Cryo-EM Image Processing

All image processing was performed using RELION 3.1 (5) and cryoSPARC v3.3.1 (6) on a GPU workstation (2 GPUs). Movie frames were aligned and summed using MotionCor2 (7). Estimation of the contrast transfer function (CTF) was performed using CTFFIND4 (8).

For WT TIA-1 sPLD fibrils, after selection based on maximum resolution and figure of merit, 4,054 good micrographs remained for further image processing. All filaments were manually picked using the manual picking method of RELION, and 610,417 segments were extracted with an inter-box distance of 38.4 Å and a box size of 900 pixels. The initial reference-free 2D classification was performed with images downscaled to 128 pixels to speed up the calculation.

Segments that contributed to suboptimal 2D class averages were discarded. We then re-extracted the selected segments without downscaling them, and with a smaller box size of 450 pixels. The segments were imported into cryoSPARC for initial model building using an ab-initio reconstruction of helical refinement. Using the de novo initial model low-pass filtered to 15 Å, we performed 3D classification with five classes and selected 56,747 segments contributing to the best 3D class. A final 3D auto-refinement converged on a helical twist of 179.37° and a helical rise of 2.38 Å. To avoid a bimodal tilt distribution during 3D classification and auto-refinement, the flags –helical_keep_tilt_prior_fixed, –limit_tilt 70, and sigma tilt and psi values of 1 were used with a starting healpix order of 5 or 0.9375° (9). Post-processing was performed to sharpen the map with a B-factor of –48.16 Å^2. Based on the gold-standard Fourier Shell Correlation (FSC) of 0.143 criteria, the overall resolution was reported to be 3.36 Å.

We noticed that WT TIA-1 sPLD fibrils exhibited a flat polymorph in addition to the twisted form. We attempted helical reconstruction and single-particle analysis of the flat polymorph. However, the flat fibrils adopted a strong preferred orientation, with their flat surfaces lying on the grid. As a result, images perpendicular to the flat surface (i.e., side-on views) were scarcely present, preventing reliable 3D reconstruction.

For G355R TIA-1 sPLD fibrils, after the image selection, 5,735 good micrographs remained for further image processing. All filaments were manually picked using the manual picking

method of RELION, and 341,191 segments were extracted with an inter-box distance of 38.4 Å and a box size of 900 pixels. The initial reference-free 2D classification was performed with images downscaled to 128 pixels to speed up the calculation. Segments that contributed to suboptimal 2D class averages were discarded. We then re-extracted the selected segments without downscaling them, and with a smaller box size of 450 pixels. The segments were imported into cryoSPARC for initial model building using an ab-initio reconstruction of helical refinement. Using the de novo initial model low-pass filtered to 10 Å, we performed several rounds of 3D classification with five classes and selected 45,382 segments contributing to the best 3D class. A final 3D auto-refinement converged on a helical twist of 179.29° and a helical rise of 2.38 Å. To avoid a bimodal tilt distribution during 3D classification and auto-refinement, the flags –helical_keep_tilt_prior_fixed, –limit_tilt 70, and sigma tilt and psi values of 1 were used with a starting healpix order of 5 or 0.9375° (9), but the final 3D auto-refinement was performed without the flags and with sigma tilt and psi values of 3. Post-processing was performed to sharpen the map with a B-factor of –40.04 Å^2. Based on the gold-standard Fourier Shell Correlation (FSC) of 0.143 criteria, the overall resolution was reported to be 3.10 Å.

The FSC curve for cryo-EM maps exhibited a sharp peak between 0.2 and 0.3 Å^{-1}. This is attributed to the fact that the pitch of the amyloid fibrils is 4.8 Å, resulting in a corresponding sharp peak at 0.21 Å^{-1}, the reciprocal of 4.8 Å, in the FSC curve. Additionally, the helical

refinement of the amyloid fibrils was achieved by setting the box size to 450 Å³, contributing to the observed noise in the FSC curve. To mitigate this noise and focus on the core part of the amyloid fibrils, refinement was performed using a reduced box size of 256 Å³, yielding estimated resolutions of 3.31 Å for the WT and 3.00 Å for G355R (Supplementary Figure 4b, d). Based on these results, we consider the map resolution to be reasonable.

Atomic model building and refinement

Coot 0.9.8.5 (10), Phenix 1.19.2-4158-000 (11), UCSF Chimera 1.10.1 (12), and PyMOL 2.4.0 (http://www.pymol.org) were used for modeling, refinement and visualization. For a model building of WT or G355R TIA-1 fibrils, various segments from the WT or G355R TIA-1 sPLD sequence were used with different orientations of their termini. However, only the 336-360 residue region for WT and the 334-349 residue region for G355R allowed us to construct an atomic model which could explain the reconstructed density map with the validity of the backbone dihedral angles.

For WT TIA-1 sPLD fibrils, an initial model was built in Coot using large side chains of W339 and W353 as a guide. The kinked segment centered on G343 and the region of $_{352}$PWMGP$_{356}$ were deviated from the general β-strand conformations, making it hard to identify

the residues and construct the models, we carefully built the models with reference to the Ramachandran plot. The single-chain model was copied to generate a three-layer model and subjected to iterative real-space refinement in PHENIX. The final model was validated using MolProbity (13).

For G355R TIA-1 sPLD fibrils, an initial model was built in Coot using large side chains of W339 and F344 as a guide. The single-chain model was copied to generate a three-layer model and subjected to iterative real-space refinement in PHENIX. The final model was validated using MolProbity.

The solvation free energies for amyloid fibril structures were calculated with the Amyloid Illustrator (https://srv.mbi.ucla.edu/AmyloidAtlas/Illustrator/). A detailed method has been described previously (14, 15). Ramachandran plots were drawn by PyRAMA (https://github.com/gerdos/PyRAMA) with some modification.

MD simulation and 3D-RISM calculation

Initial structures of proteins were taken from PDB ID 9KTY for WT and 9KTZ for G355R. Amber20 package (16) was used for MD simulation for structural sampling of monomer, pentamer and hexamer of amyloid fibrils. Amber ff99SB force field (17) and OPC parameters (18) were employed for proteins

and water, respectively. Sampling was performed at 100 ns for each system following 1-ns equilibration simulation. The simulations were conducted at 300 K and 1 bar in the NPT ensemble under the MC barostat and Langevin thermostat. Time step was set to 0.002 ps, and the SHAKE method (19) was applied for hydrogen constrain. A periodic boundary box was prepared with a margin of 15 Å from the end of each edge protein. For the 3D-RISM calculation (20, 21), 100 snapshots taken from the MD trajectories every 1 ns. All the water molecules were stripped from the snapshots. The 3D-RISM equation was solved by coupling with the Kovalenko-Hirata closure (22) using in-house 3D-RISM program code (23, 24). TIP3P water parameters (25) were used for 3D-RISM calculation with 128^3 Å^3 solvation box with 0.5 Å grid spacing.

Statistical information

Microsoft Excel and Python matplotlib library were used for statistical analysis and data visualization. ThT assay and fibril disassembly assay were performed at least twice with three or four technical replicates. Data are presented as mean ± standard deviation (s.d.). Multiple comparisons were evaluated using one-way ANOVA followed by a post hoc Tukey honest significant difference test (https://www.statskingdom.com/180Anova1way.html). The p-values are shown in the figure and in the corresponding legend. A p-value less than 0.05 was considered statistically significant.

Data Availability

The atomic coordinates and structure factors have been deposited in the Protein Data Bank, www.pdb.org (PDB/EMDB ID codes: 9KTY/EMD-62570 for WT and 9KTZ/EMD-62571 for G355R).

Code Availability

In-house 3D-RISM code is implemented in the RISMiCal program package. It can be shared upon reasonable request to the authors. The RISMiCal program package is being prepared for open source.

SI References

1. F. E. Loughlin, *et al.*, Tandem RNA binding sites induce self-association of the stress granule marker protein TIA-1. *Nucleic Acids Res* **49**, 2403–2417 (2021).
2. N. Sekiyama, *et al.*, ALS mutations in the TIA-1 prion-like domain trigger highly condensed pathogenic structures. *Proc Natl Acad Sci U S A* **119**, 1–12 (2022).
3. D. N. Mastronarde, Automated electron microscope tomography using robust prediction of specimen movements. *J Struct Biol* **152** (2005).
4. K. Yonekura, S. Maki-Yonekura, H. Naitow, T. Hamaguchi, K. Takaba, Machine learning-based real-time object locator/evaluator for cryo-EM data collection. *Commun Biol* **4**, 1–8 (2021).
5. J. Zivanov, *et al.*, New tools for automated high-resolution cryo-EM structure determination in RELION-3. *Elife* **7**, 1–22 (2018).
6. A. Punjani, J. L. Rubinstein, D. J. Fleet, M. A. Brubaker, CryoSPARC: Algorithms for rapid unsupervised cryo-EM structure determination. *Nat Methods* **14**, 290–296 (2017).
7. S. Q. Zheng, *et al.*, MotionCor2: Anisotropic correction of beam-induced motion for improved cryo-electron microscopy. *Nat Methods* **14**, 331–332 (2017).
8. A. Rohou, N. Grigorieff, CTFFIND4: Fast and accurate defocus estimation from electron micrographs. *J Struct Biol* **192**, 216–221 (2015).
9. C. Glynn, *et al.*, Cryo-EM structure of a human prion fibril with a hydrophobic, protease-resistant core. *Nat Struct Mol Biol* **27**, 417–423 (2020).
10. P. Emsley, B. Lohkamp, W. G. Scott, K. Cowtan, Features and development of Coot. *Acta Crystallogr D Biol Crystallogr* **66**, 486–501 (2010).
11. D. Liebschner, *et al.*, Macromolecular structure determination using X-rays, neutrons and electrons: Recent developments in Phenix. *Acta Crystallogr D Struct Biol* **75**, 861–877 (2019).
12. E. F. Pettersen, *et al.*, UCSF Chimera - A visualization system for exploratory research and analysis. *J Comput Chem* **25**, 1605–1612 (2004).
13. C. J. Williams, *et al.*, MolProbity: More and better reference data for improved all-atom structure validation. *Protein Science* **27**, 293–315 (2018).
14. D. Eisenberg, A. D. McLachlan, Solvation energy in protein folding and binding. *Nature* **319**, 199–203 (1986).
15. M. R. Sawaya, M. P. Hughes, J. A. Rodriguez, R. Riek, D. S. Eisenberg, The expanding amyloid family: Structure, stability, function, and pathogenesis. *Cell* **184**, 4857–4873 (2021).
16. D. A. Case, *et al.*, AMBER2020, university of California, San Fransisco. *J. Amer. Chem. Soc* **142**, 3823–3835 (2020).
17. V. Hornak, *et al.*, Comparison of multiple Amber force fields and development of improved protein backbone parameters. *Proteins: Structure, Function, and Bioinformatics* **65**, 712–725

(2006).

18. P. S. Shabane, S. Izadi, A. V. Onufriev, General Purpose Water Model Can Improve Atomistic Simulations of Intrinsically Disordered Proteins. *J Chem Theory Comput* **15**, 2620–2634 (2019).
19. J. P. Ryckaert, G. Ciccotti, H. J. C. Berendsen, Numerical integration of the cartesian equations of motion of a system with constraints: molecular dynamics of n-alkanes. *J Comput Phys* **23**, 327–341 (1977).
20. D. Beglov, B. Roux, Solvation of complex molecules in a polar liquid: An integral equation theory. *Journal of Chemical Physics* **104**, 8678–8689 (1996).
21. A. Kovalenko, F. Hirata, Three-dimensional density profiles of water in contact with a solute of arbitrary shape: A RISM approach. *Chem Phys Lett* **290**, 237–244 (1998).
22. A. Kovalenko, F. Hirata, Potentials of mean force of simple ions in ambient aqueous solution. II. Solvation structure from the three-dimensional reference interaction site model approach, and comparison with simulations. *Journal of Chemical Physics* **112**, 10403–10417 (2000).
23. Y. Maruyama, F. Hirata, Modified anderson method for accelerating 3D-RISM calculations using graphics processing unit. *J Chem Theory Comput* **8**, 3015–3021 (2012).
24. Y. Maruyama, *et al.*, Massively parallel implementation of 3D-RISM calculation with volumetric 3D-FFT. *J Comput Chem* **35**, 1347–1355 (2014).
25. W. L. Jorgensen, J. Chandrasekhar, J. D. Madura, R. W. Impey, M. L. Klein, Comparison of simple potential functions for simulating liquid water. *J Chem Phys* **79**, 926–935 (1983).

Supplementary Figure 1 to 10

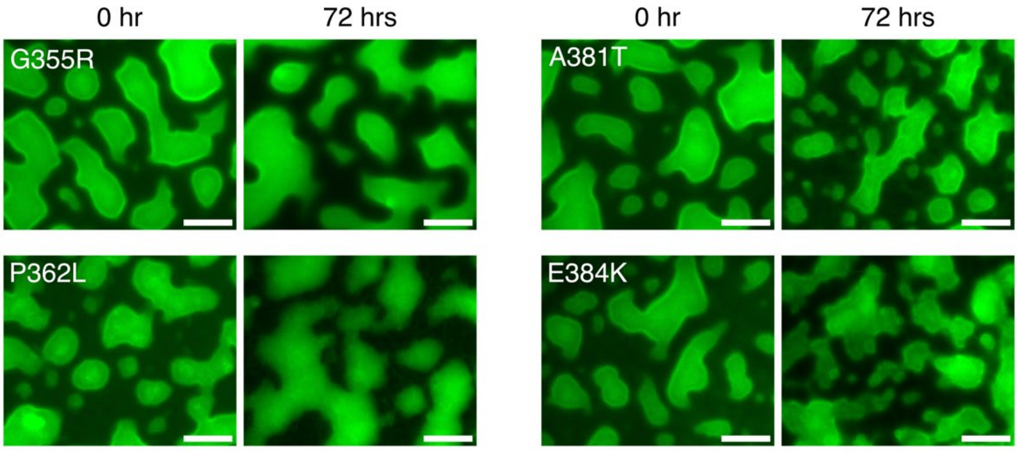

Supplementary Fig. 1. The full-length TIA-1 condensates for mutants.

Fluorescence microscopic images of the full-length TIA-1 condensates for G355R, P362L, A381T and E384K in the presence of the single-stranded DNA TC5. The left panel shows the initial image at 0 hr, and the right panel shows the image after 72 hrs of incubation. The sample contains 10 μM full-length TIA-1 and 0.05 μM TC5 in 50 mM HEPES, pH 7.5, 150 mM NaCl, 1 mM DTT. Condensates were visualized with the addition of 10 μM Thioflavin T. Scale bar, 10 μm.

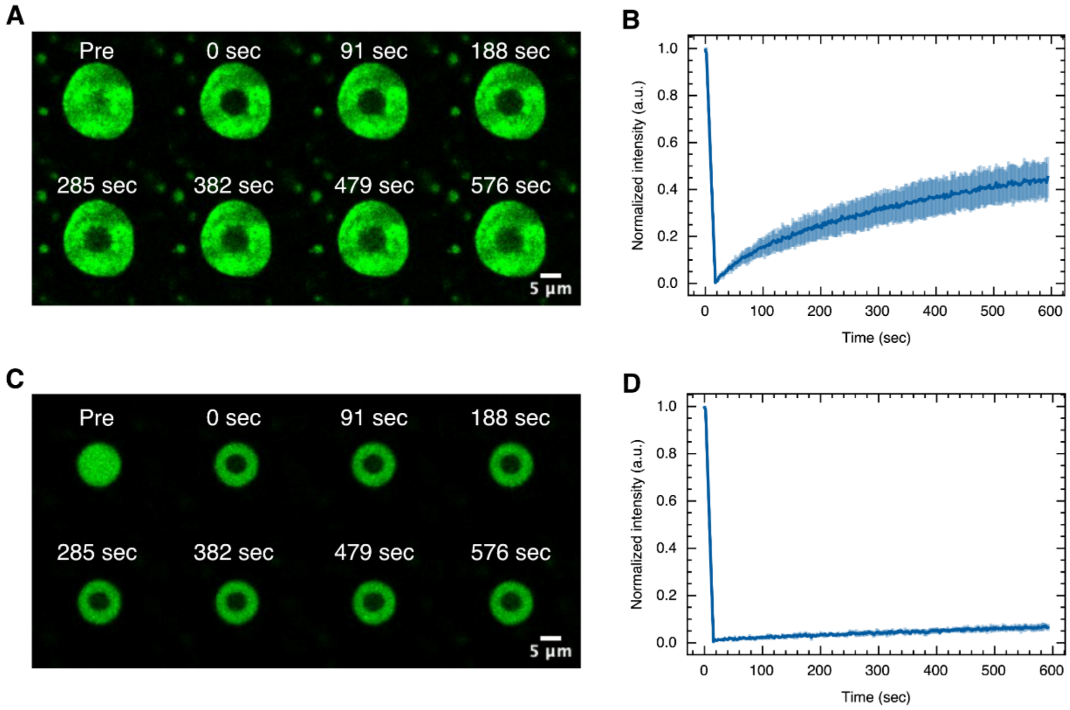

Supplementary Fig. 2. FRAP analysis of TIA-1 phase-separated condensates.

A, C) Time-lapse fluorescence images at each time point of the full-length WT condensates (A) and the sPLD WT condensates (C) after photobleaching.

B, D) Fluorescence recovery curves of the full-length WT condensates (B), and the WT sPLD condensates (D). The fluorescence intensity was normalized between 0 and 1. Data represent the mean ± standard deviation (n = 7). Scale bar, 5 μm.

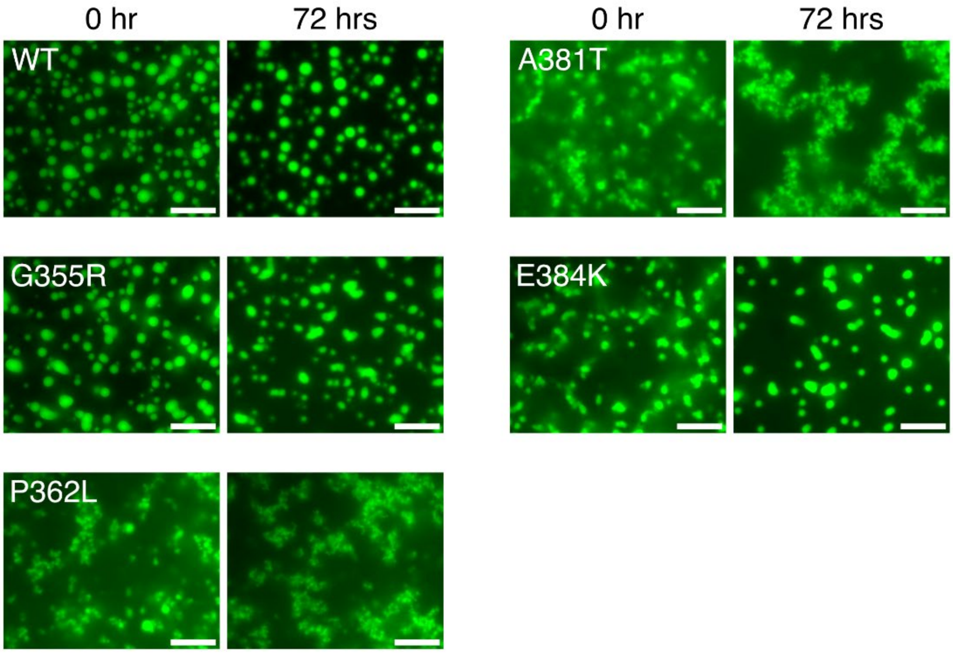

Supplementary Fig. 3. The TIA-1 sPLD condensates for mutants.

Fluorescence microscopic images of the TIA-1 sPLD condensates for G355R, P362L, A381T and E384K. The corresponding WT images (Fig. 1f,g) are shown for comparison. The left panel shows the initial image of each sample at 0 hr, and the right panel shows the image after 72 hrs of incubation. The sample contains 25 μM TIA-1 sPLD in 50 mM HEPES, pH 7.5, 150 mM NaCl, 1 mM DTT. Condensates were visualized with the addition of 10 μM Thioflavin T. Scale bar, 10 μm.

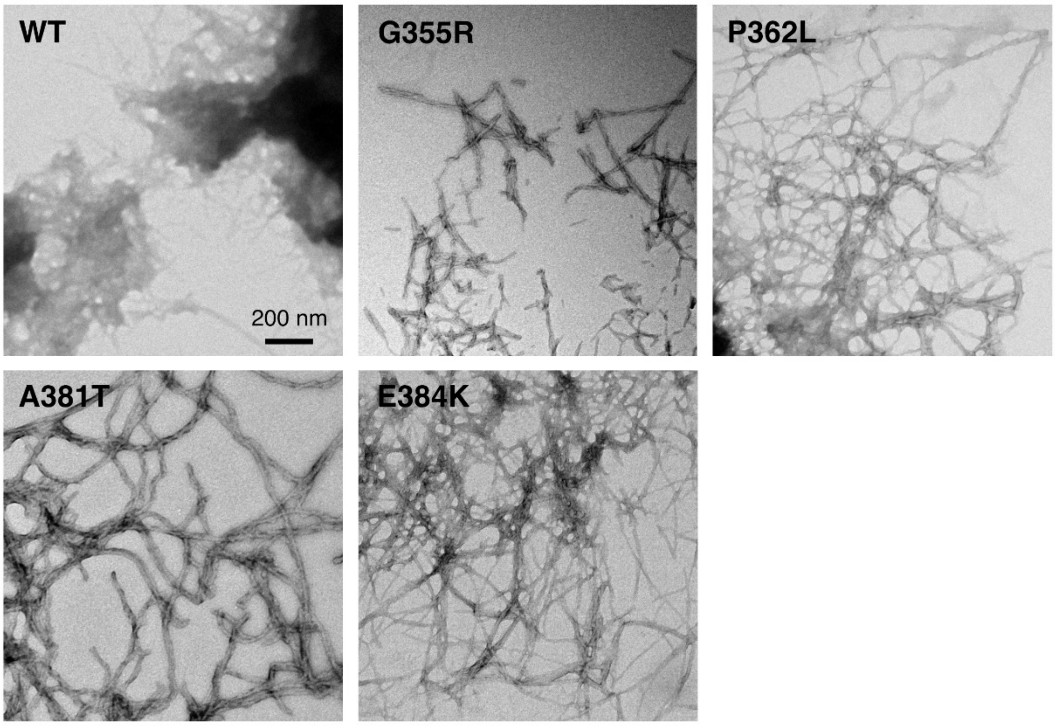

Supplementary Fig. 4. Negative-staining TEM images of WT and mutant fibrils

Representative negative-staining TEM images of WT and mutant (G355R, P362L, A381T, and E384K) TIA-1 sPLD fibrils. Scale bar, 200 nm.

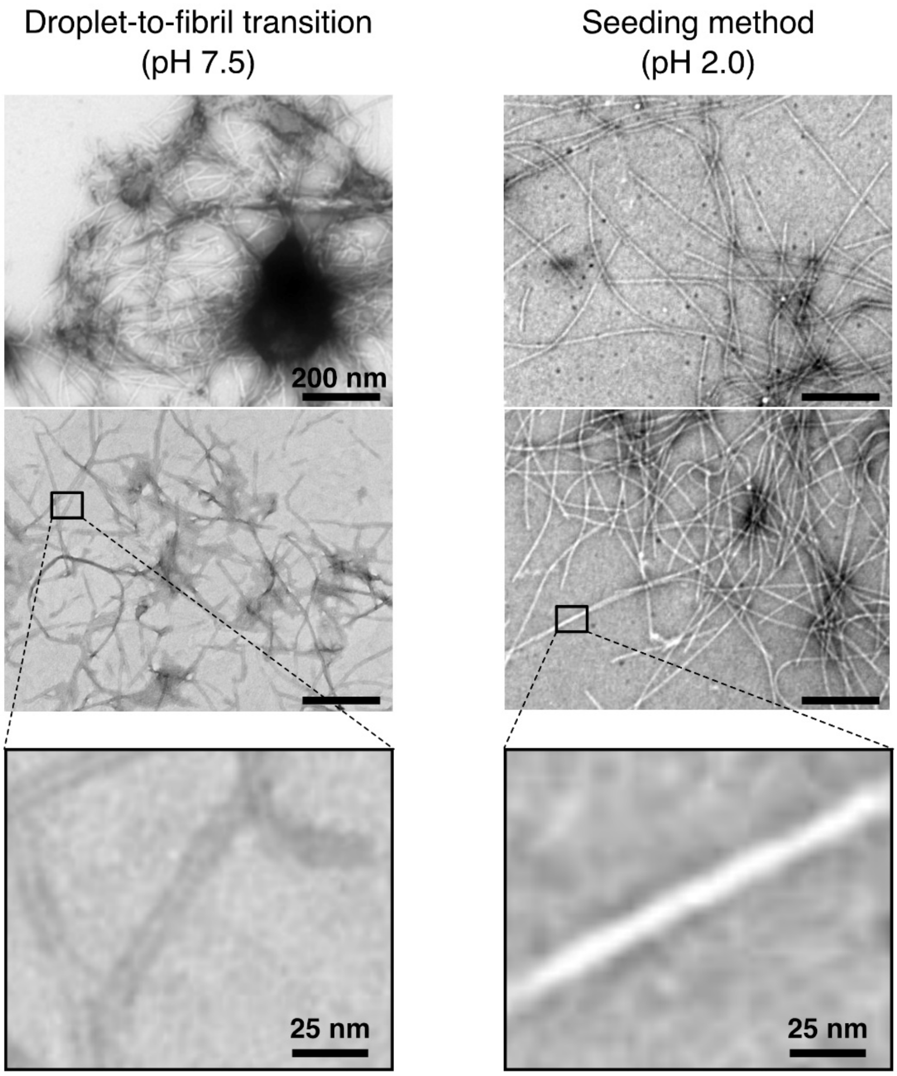

Supplementary Fig. 5. Comparison of amyloid fibril morphologies between fibrils formed by condensate maturation and those by the seeding method.

Negative-staining TEM images of WT TIA-1 sPLD fibrils formed by condensate maturation and by the seeding method. The lower images present a magnified view of the black box highlighted in the central image.

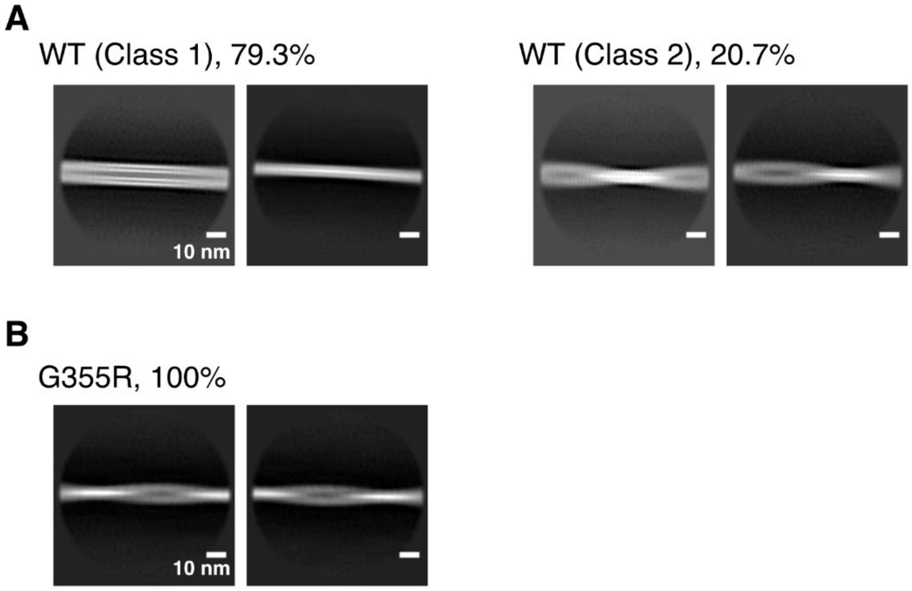

Supplementary Fig. 6. Structural types of WT and G355R TIA-1 sPLD fibrils revealed by cryo-EM.

A) WT TIA-1 sPLD fibrils had two structural types: flat fibrils (Class 1) and twisted fibrils (Class 2). Flat fibrils accounted for 79.3%, while twisted fibrils accounted for 20.1%.

B) G355R TIA-1 sPLD fibrils had one structural type of twisted fibrils.

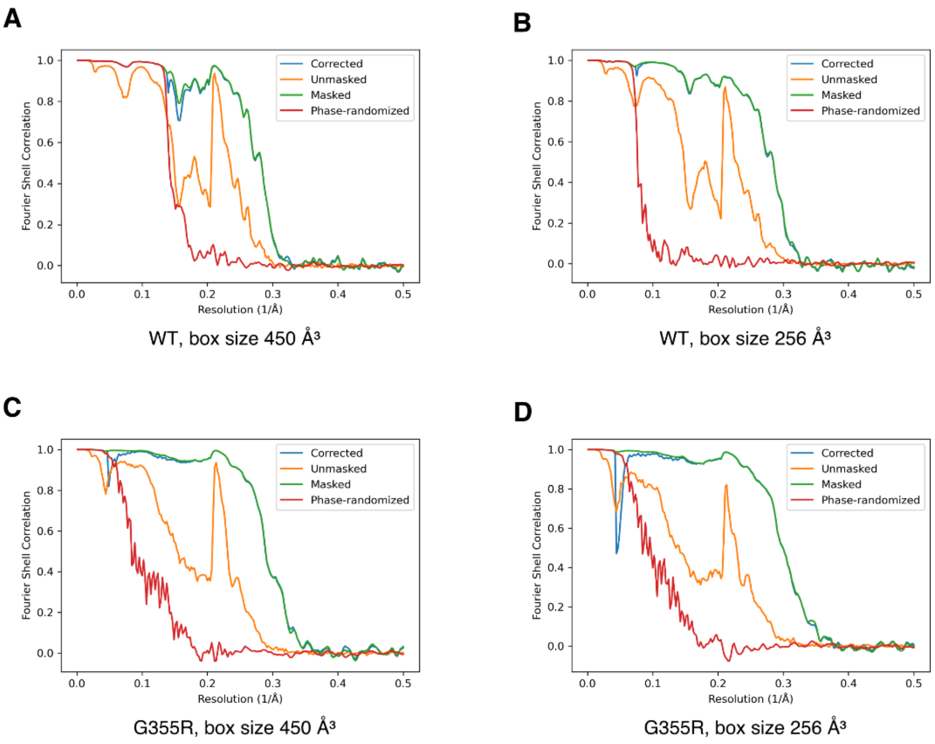

Supplementary Fig. 7. Gold-standard Fourier shell correlation (FSC) curves for WT (A, B) and G355R (C, D).

The FSC curves with mask, without mask, phase randomization, and the final corrected FSC curves are shown in green, orange, red, and blue, respectively. Box size were 450 $Å^3$ for (A) and (C), and 256 $Å^3$ for (B) and (D).

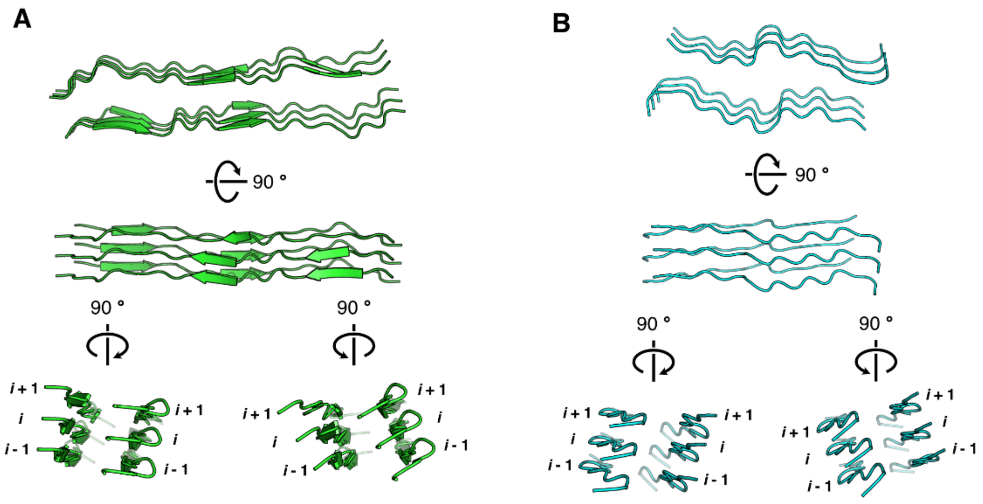

Supplementary Fig. 8. Ribbon model diagram of the β-strands in WT (A) and G355R (B) TIA-1 sPLD fibril structures.

The model shows a three-layer β-strand structures comprising two protofilaments.

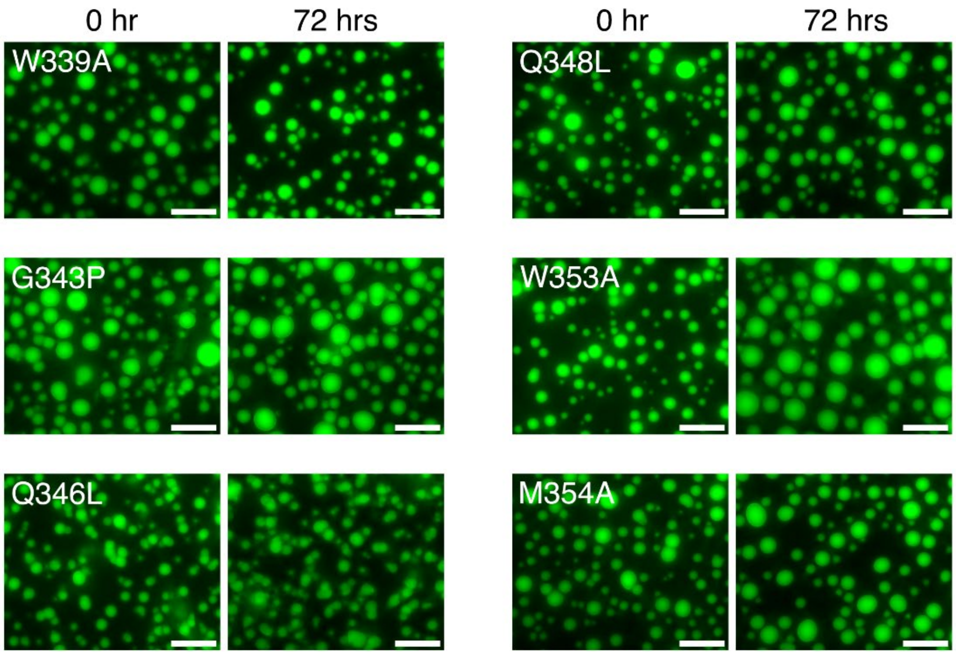

Supplementary Fig. 9. The TIA-1 sPLD condensates for mutants.

Fluorescence microscopic images of the TIA-1 sPLD condensates for W339A, G343P, Q346L, Q348L, W353A, and M354A. The left panel shows the initial image of each sample at 0 hr, and the right panel shows the image after 72 hrs of incubation. The sample contains 25 μM TIA-1 sPLD in 50 mM HEPES, pH 7.5, 150 mM NaCl, 1 mM DTT. Condensates were visualized with the addition of 10 μM Thioflavin T. Scale bar, 10 μm.

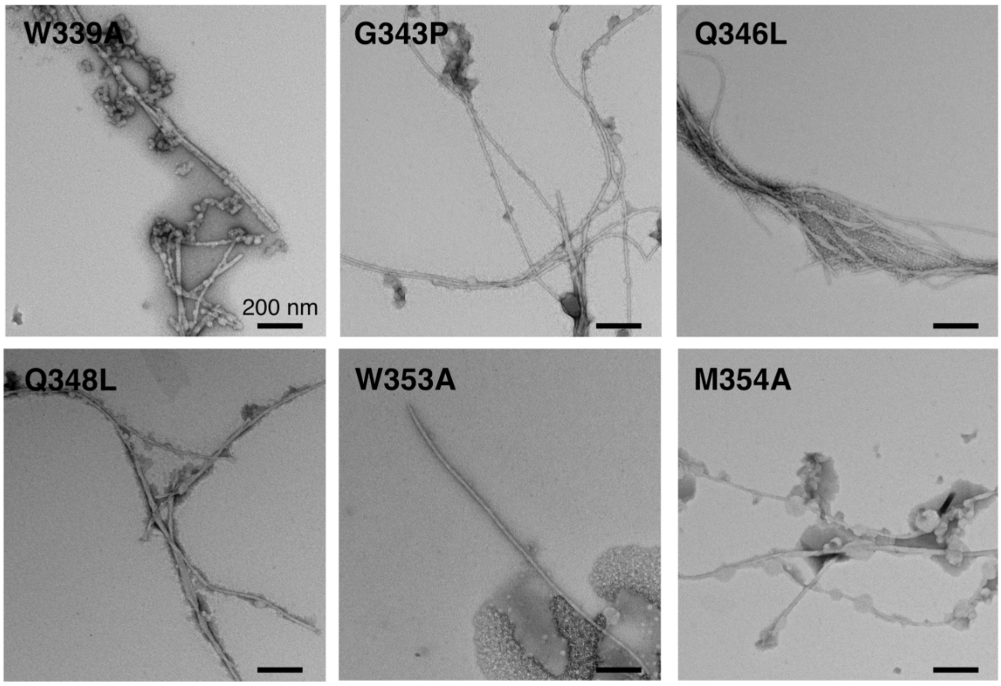

Supplementary Fig. 10. Negative-staining TEM images of mutant TIA-1 sPLD fibrils

Representative negative-staining TEM images of mutant (W339A, G343P, Q346L, Q348L, W353A, and M354A) TIA-1 sPLD fibrils. Scale bar, 200 nm.
